# The 2-methylcitrate cycle is implicated in the detoxification of propionate in *Toxoplasma gondii*

**DOI:** 10.1111/mmi.12139

**Published:** 2013-01-11

**Authors:** Julien Limenitakis, Rebecca D Oppenheim, Darren J Creek, Bernardo J Foth, Michael P Barrett, Dominique Soldati-Favre

**Affiliations:** 1 Department of Microbiology and Molecular Medicine, Faculty of Medicine, University of GenevaCMU 1 Rue Michel Servet, 1211, Geneva, Switzerland; 2 Wellcome Trust Center for Molecular Parasitology, University of Glasgow120 University Place, Glasgow, G12 8TA, UK; 3 Department of Biochemistry and Molecular Biology, Bio21 Molecular Science and Biotechnology Institute, University of MelbourneFlemington Rd, Parkville, Victoria, 3010, Australia; 4 Wellcome Trust Sanger InstituteHinxton, UK

## Abstract

*Toxoplasma gondii* belongs to the coccidian subgroup of the Apicomplexa phylum. The Coccidia are obligate intracellular pathogens that establish infection in their mammalian host via the enteric route. These parasites lack a mitochondrial pyruvate dehydrogenase complex but have preserved the degradation of branched-chain amino acids (BCAA) as a possible pathway to generate acetyl-CoA. Importantly, degradation of leucine, isoleucine and valine could lead to concomitant accumulation of propionyl-CoA, a toxic metabolite that inhibits cell growth. Like fungi and bacteria, the Coccidia possess the complete set of enzymes necessary to metabolize and detoxify propionate by oxidation to pyruvate via the 2-methylcitrate cycle (2-MCC). Phylogenetic analysis provides evidence that the 2-MCC was acquired via horizontal gene transfer. In *T. gondii* tachyzoites, this pathway is split between the cytosol and the mitochondrion. Although the rate-limiting enzyme 2-methylisocitrate lyase is dispensable for parasite survival, its substrates accumulate in parasites deficient in the enzyme and its absence confers increased sensitivity to propionic acid. BCAA is also dispensable in tachyzoites, leaving unresolved the source of mitochondrial acetyl-CoA.

## Introduction

*Toxoplasma gondii* is an obligate intracellular parasite that belongs to the phylum of Apicomplexa. This pathogen is responsible for one of the most widespread parasitic infections of humans and other warm-blooded animals (Elmore *et al*., 2010). The large variety of hosts and cell types that sustain *T. gondii* growth reflects the considerable metabolic plasticity of this parasite (Seeber *et al*., 2008; Polonais and Soldati-Favre, 2010). Host cell invasion leads to the formation of a unique, non-phagosomal, parasitophorous vacuole inside which the parasite proliferates (Boyle and Radke, 2009). *T. gondii* has evolved different strategies in order to acquire nutrients such as lipids, by scavenging them from the host (Coppens *et al*., 2000; Charron and Sibley, 2002). *De novo* fatty acid synthesis also occurs through an ACP-dependent FAS II pathway hosted in the apicoplast, a multi-membrane relict plastid resulting from a secondary endosymbiotic event (McFadden *et al*., 1996; Kohler *et al*., 1997; Fleige *et al*., 2010). Additionally, *T. gondii* possesses a typical FAS I eukaryotic multifunctional enzyme localized in the cytosol (Mazumdar and Striepen, 2007) and several elongases in the endoplasmic reticulum (Ramakrishnan *et al*., 2012). The pyruvate dehydrogenase (PDH) complex of Plasmodium and Toxoplasma is targeted exclusively to the apicoplast for FASII (Foth *et al*., 2005; Fleige *et al*., 2007), while the source of carbon to produce acetyl-CoA for FASI remains uncertain. Interestingly, the coccidian subgroup of the Apicomplexa has retained the capacity to degrade the branched-chain amino acids (BCAA) leucine, isoleucine and valine, which offers one plausible source of acetyl-CoA in the mitochondrion (Seeber *et al*., 2008). Importantly, degradation of BCAA gives rise to the concomitant production of propionyl-CoA, a cytotoxic metabolite that inhibits cell growth (Horswill *et al*., 2001; Brock and Buckel, 2004; Schwab *et al*., 2006). Commonly in mammalian cells, the methyl-malonyl-CoA pathway serves for the detoxification of propionyl-CoA but is, however, absent in the Apicomplexa. In enterobacteria, mycobacteria, yeast and soil-dwelling filamentous fungi, the 2-methylcitrate cycle (2-MCC) is used as an alternative pathway for the detoxification process (Uchiyama *et al*., 1982; Pronk *et al*., 1994). This pathway involves five enzymes that allow conversion of propionate to pyruvate. In mycobacteria the 2-MCC is implicated in detoxification but also fulfils a minor role of carbon conversion in an anaplerotic fashion (Upton and McKinney, 2007). In *Aspergillus fumigatus*, deletion of the gene coding for the key enzyme methylcitrate synthase (PrpC) leads to accumulation of propionyl-CoA along with a growth retardation and reduction of secondary metabolite synthesis derived from polyketides (Zhang *et al*., 2004). In *Rhodobacter sphaeroides* and in *Aspergillus niger*, propionyl-CoA was shown to inhibit the PDH (Brock and Buckel, 2004; Maerker *et al*., 2005). Furthermore, 2-methylcitrate has also been shown to be toxic especially by inhibiting the NADP-dependent isocitrate dehydrogenase (Brock, 2005). In *Mycobacterium tuberculosis*, a mutant lacking *prpDC* is unable to grow on propionate media or in murine bone marrow-derived macrophages but no alteration of virulence is observed in mice (Munoz-Elias *et al*., 2006). The importance of 2-MCC in propionate detoxification has also been demonstrated in *Mycobacterium smegmatis* (Upton and McKinney, 2007).

Here we provide evidence for the presence of a functional 2-MCC in *T. gondii* that was acquired via horizontal gene transfer by an ancestor of the Alveolata. The 2-MCC has been preserved in cyst-forming parasites that infect the intestinal tract of animals but was lost in Apicomplexans that lack the BCAA degradation and FAS I pathways (Seeber *et al*., 2008). In *T. gondii*, the 2-MCC is distributed between the mitochondrion and cytosol. Ablation of the 2-methylisocitrate lyase gene (*TgPrpB*) established that 2-MCC is dispensable for parasite propagation *in vitro* and *in vivo*. Moreover, the disruption of the branch-chain aminotransferase (*TgBCAT*) gene revealed that the BCAA degradation is non-essential in tachyzoites and hence not the major source of propionate for the 2-MCC. Parasites lacking a functional 2-MCC display increased sensitivity to propionate pointing to a role in detoxifying this metabolic product, which can be encountered in the host intestinal environment.

## Results

### The complete set of genes coding for the 2-methylcitrate cycle is present in Coccidia

Mining of the *T. gondii* genome database revealed the presence of all metabolic enzymes required for the 2-MCC (Seeber *et al*., 2008), whereas the methyl-malonyl-CoA pathway implicated in detoxification of propionyl-CoA in mammalian cells is absent in Apicomplexa.

**Fig. 1:**
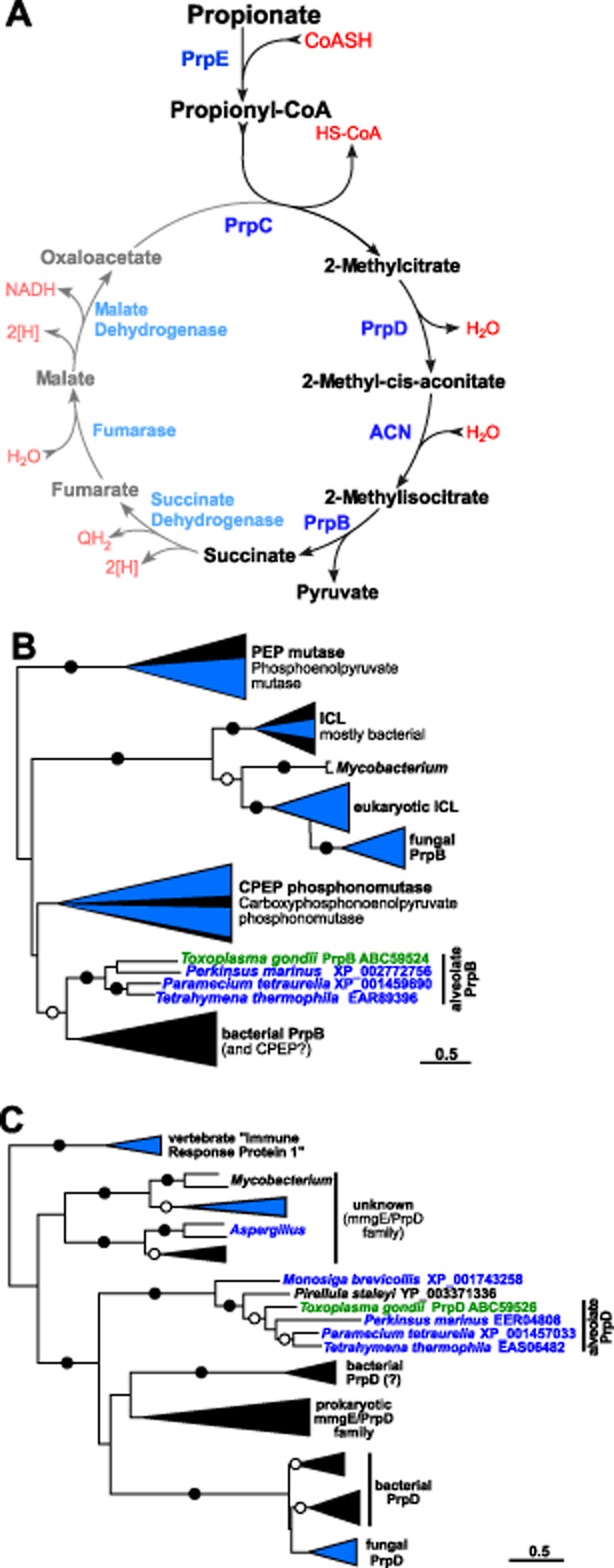
The 2-methylcitrate cycle is present and conserved in Coccidia. A. Scheme of the 2-methylcitrate cycle and the C4 part of the tricarboxylic acid cycle forming the 2-methylcitrate cycle. B and C. Simplified unrooted PhyML maximum-likelihood (ML) phylogenies of PrpB and PrpD protein sequences respectively. The complete trees showing all branches and nodes are provided as Figs S1 and S2. Filled circles on the branches indicate bootstrap support of at least 90% in both ML and neighbour-joining (NJ) analyses, whereas open circles indicate bootstrap support of at least 90% in either ML or NJ analysis (100 pseudoreplicates). Sequences from eukaryotic organisms are represented in blue and green, whereas black represents prokaryotic sequences.

The 2-MCC allows the α-oxidation of propionate through five specific enzymatic steps (Tabuchi and Hara, 1974; Uchiyama *et al*., 1982), including the activation of propionate to propionyl-CoA mediated by the propionyl-CoA synthetase (PrpE). Oxaloacetate and propionyl-CoA are then condensed into 2-methylcitrate by the 2-methylcitrate synthase (PrpC) followed by conversion to 2-methyl-cis-aconitate by the action of a 2-methylcitrate dehydratase (PrpD). The subsequent hydratase reaction is catalysed by an aconitase (Acn), resulting in a 2-methylisocitrate molecule, which is finally cleaved by the 2-methylisocitrate lyase (PrpB) into pyruvate and succinate. This last detoxification step in conjunction with enzymes of the C4 part of the TCA cycle allows bacteria and fungi to use propionate as a carbon source (Brock *et al*., 2000; Brock *et al*., 2002) (Fig. 1A).

Investigating the distribution of 2-MCC genes in apicomplexan genomes uncovered significant differences between various parasites of this phylum. Only the members of the coccidian branch, namely *T. gondii*, *Neospora caninum* and *Eimeria tenella*, possess genes coding for enzymes of the 2-MCC. In contrast, other branches of the phylum, such as Haemosporidia (e.g. *Plasmodium*), Piroplasmida (e.g. *Theileria*) and *Cryptosporidium*, lack these genes (Table 1).

**Table 1:** Methylcitrate cycle genes in apicomplexan parasites and other alveolate protists

EC Number	Abbreviations	Enzyme name	Mitochondrial targeting signal probability (MitoprotII)	*Tg*	
6.2.1.17	PrpE	Propionyl-CoA synthetase	0.9289	TGME49_032580	
2.3.3.5	PrpC	2-Methylcitrate synthase	0.9521	ABC59525	
4.2.1.79	PrpD	2-Methylcitrate dehydratase	0.1982	ABC59526	
4.1.3.30	PrpB	2-Methylisocitrate lyase	0.0778	ABC59524	
4.2.1.99	Acn	Aconitate hydratase	0.9955	AAT68238	
–		Dicarboxylic acid transporter	0.6321	TGME49_074060	

Abbreviations	*Nc*	*Et*	*Pf*	*Cp*	*Tp*
PrpE	NCLIV_032540	ETH_00007195	–	–	–
PrpC	NCLIV_024780	ETH_00026655	–	–	–
PrpD	NCLIV_013410	ETH_00015105	–	–	–
PrpB	NCLIV_000780	Contig 1606+713	–	–	–
Acn	NCLIV_046260	ETH_00020300	PF13_0229	–	TP01_1050
	NCLIV_033680	ETH_00021115	PF08_0031	cgd1_600	TP02_0450

Abbreviations	*Bb*	*Pm*	*Pt*	*Tt*	
PrpE	–	–	XP_001460124	XP_001025576	
PrpC	–	XP_002787782	XP_001447471	XP_001023516	
PrpD	–	EER04808	XP_001457033	EAS06482	
PrpB	–	XP_002772756	XP_001459690	EAR89396	
Acn	BBOV_III011790	XP_002765934	XP_001451848	XP_001016756	
	BBOV_III005160	XP_002768944	XP_001431858	XP_001031896	

*Tg*, *Toxoplasma gondii*; *Nc*, *Neospora caninum*; *Et*, *Eimeria tenella*; *Pf*, *Plasmodium falciparum*; *Cp*, *Cryptosporidium parvum*; *Tp*, *Theileria parva*; *Bb*, *Babesia bovis*; *Pm*, *Perkinsus marinus*; *Pt*, *Paramecium tetraurelia*; *Tt*, *Tetrahymena thermophila*.

To investigate the apparent restriction of the 2-MCC within the Apicomplexa to Coccidia and to better understand its evolutionary origin we conducted phylogenetic analyses. Both PrpB and PrpD phylogenies (Figs 1B and C) revealed a very close relationship of the apicomplexan genes with those of ciliates (*Tetrahymena* and *Paramecium*) and *Perkinsus*, a plausible scenario since apicomplexans, ciliates and *Perkinsus* all belong to the monophyletic group Alveolata. Furthermore, for both PrpB and PrpD the phylogenetic trees revealed distant relationships of the alveolate 2-MCC genes with their fungal homologues and established closer links to bacterial sequences instead.

The PrpB phylogeny (Figs 1B and S1) clearly shows that the fungal PrpB proteins are most closely related to eukaryotic isocitrate lyases (ICL) including the fungal ICLs, from which they most likely derive as also observed elsewhere (Muller *et al*., 2011). In contrast, the maximum-likelihood tree places the alveolate PrpB genes together with prokaryotic PrpB-like sequences and shows that they are only distantly related to ICLs and the fungal PrpB sequences.

For PrpD, the fungal sequences are distinctly most closely related to a group of bacterial PrpDs (Figs 1C and S2) from which they likely derive, whereas the alveolate PrpDs are not part of this cluster. Instead, the alveolate sequences form a strongly supported clade that itself is only loosely associated with a number of prokaryotic and fungal sequences that are mostly annotated as belonging to the mmgE/PrpD family. Intriguingly, the well-supported alveolate PrpD clade also includes one sequence from the planctomycete bacterium *Pirellula staleyi* and one from the choanoflagellate *Monosiga brevicollis*. Both planctomycetes and choanoflagellates live in aquatic environments and thus share habitats with alveolate protists such as ciliates and *Perkinsus*. One plausible explanation for the unexpected distribution of alveolate-like PrpD genes is therefore gene exchange by lateral transfer between aquatic planctomycetes, choanoflagellates and alveolate protists. Furthermore, the PrpD sequences of other planctomycetes (*Isosphaera pallida*, *Singulisphaera acidiphila* and *Gemmata obscuriglobus*) are more closely related to the homologues of other members of the prokaryotic mmgE/PrpD family than to those of *Pirellula* and the alveolates (data not shown), suggesting that the *Pirellula* PrpD sequence derives from an unusual gene transfer event and making it unlikely that the alveolate PrpD sequences were acquired from an ancestor of the planctomycetes.

Taken together, for both PrpB and PrpD a substantial evolutionary distance is clearly evident between the alveolate enzymes and their fungal homologues, with the exact origin of the alveolate 2-MCC sequences currently uncertain. Importantly, the well-supported close relationship of apicomplexan *PrpB* and *PrpD* genes with those of ciliates and *Perkinsus* suggests that the genes of the 2-MCC were acquired in an ancestor of the Alveolata and that they were secondarily lost over time in all apicomplexans apart from the Coccidia.

### The 2-methylcitrate cycle is split between the mitochondrion and cytosol

**Fig. 2:**
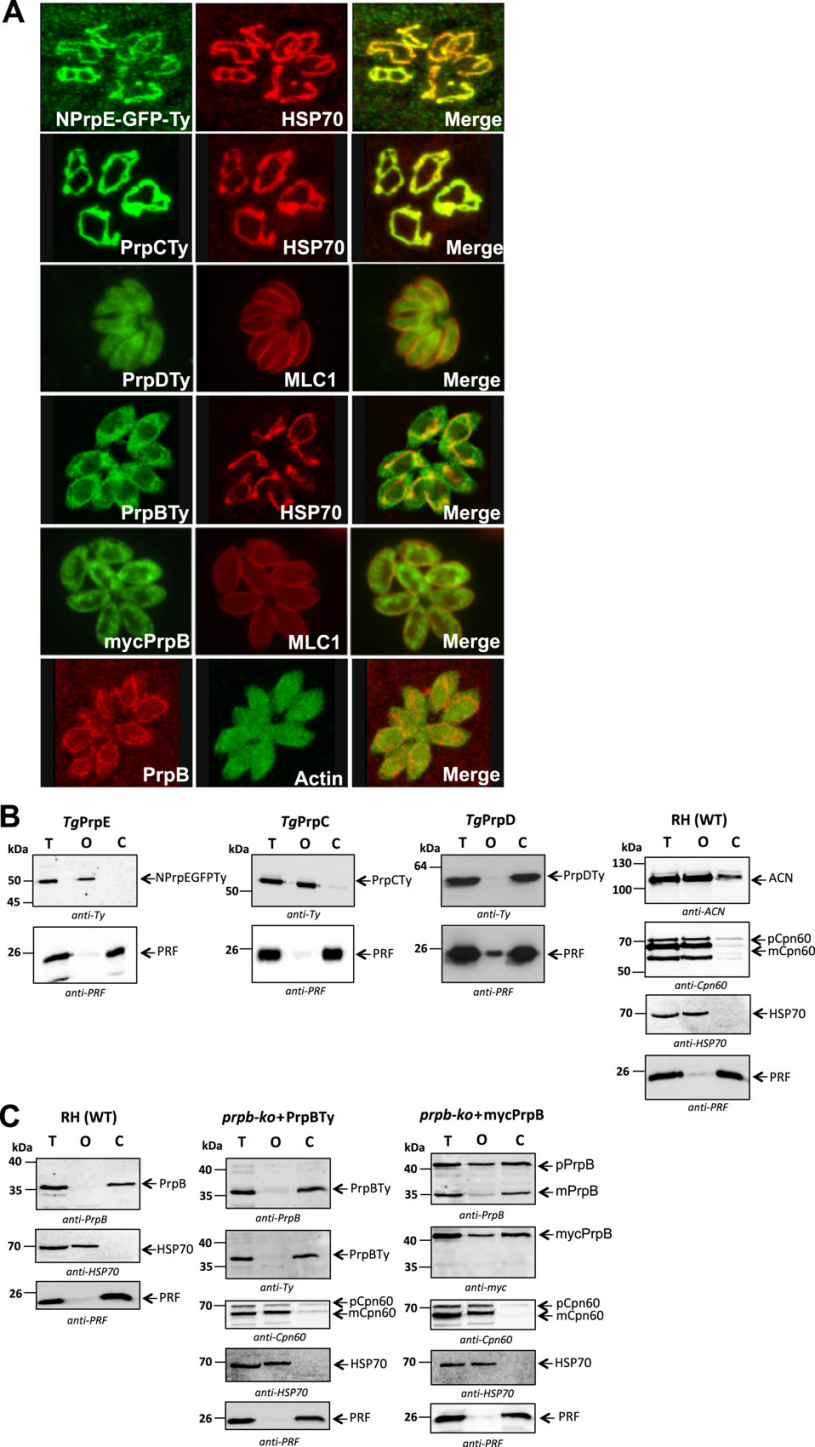
The 2-methylcitrate cycle is split between the mitochondrion and the cytosol. A. The subcellular localization of four enzymes implicated in the 2-MCC by IFA on *T. gondii* tachyzoites expressing NPrpE-GFP-Ty, PrpCTy, PrpDTy, mycPrpB or WT parasites. Anti-Ty, anti-myc and anti-actin (cytosolic marker) antibodies are labelled in green whereas anti-PrpB, anti-HSP70 (mitochondrial marker) and anti-MLC1 (pellicle marker) are labelled in red. B. Western blot analyses performed on wild-type and transgenic parasites expressing NPrpE-GFP-Ty, PrpCTy, PrpDTy following cytosolic/organellar fractionation. The Ty-tagged proteins were detected with anti-Ty antibodies and TgACN with anti-ACN antibodies. Profilin (PRF) was used as cytosolic marker. Cpn60 (apicoplast marker pCpn60, precursor; mCpn60, mature Cpn60) and HSP70 (mitochondrial marker) were used as control for organellar membrane integrity during digitonin controlled lysis. C. Western blot analyses performed on wild-type and transgenic parasites *prpb-ko*+PrpBTy and *prpb-ko*+mycPrpB following cytosolic/organellar fractionation. Endogenous PrpB, PrpB-Ty and mycPrpB were detected by anti-PrpB, anti-Ty and anti-myc respectively. PRF, Cpn60 and HSP70 are used as controls for cytosolic and organellar fractions respectively. A single representative Western blot is presented. Fractionation experiments were repeated at least three times. T, total cell lysate of tachyzoites; O, organellar fraction; C, cytosolic fraction.

The genes coding for the 2-MCC were experimentally annotated and the protein sequences were analysed for the presence of targeting signals using the TargetP 1.1- and MitoprotII- algorithms. The results summarized in Table 1 indicate a putative mitochondrial import signal for TgPrpE and TgPrpC, whereas TgPrpD and TgPrpB are predicted to be cytosolic. The aconitase was previously described to be dually targeted to the mitochondrion and apicoplast (Pino *et al*., 2007). To determine experimentally the localization of the *T. gondii* 2-MCC enzymes, stable transgenic parasites expressing epitope-tagged versions of these proteins were generated. For this purpose, the ORFs of *TgPrpC*, *TgPrpD* and *TgPrpB* were cloned into expression plasmids and C-terminally fused to a Ty1 tag (TgPrpC-Ty; TgPrpD-Ty; TgPrpB-Ty). Due to its large size, only the N-terminal fragment of PrpE (280 first amino acids) was cloned into an expression plasmid to fuse it to a C-terminal GFP-Ty tag (NTgPrpE-GFP-Ty). In addition an N-terminal myc tag fusion was generated for PrpB (myc-TgPrpB). The localization of the tagged proteins was determined by indirect immunofluorescence staining using antibodies against Ty1 or myc. NTgPrpE-GFP-Ty and TgPrpC-Ty were found inside the single tubular mitochondrion, while TgPrpD-Ty was found cytosolic as was predicted by the *in silico* analysis (Fig. 2A). TgPrpB-Ty and myc-TgPrpB were found to be largely cytosolic, although a small fraction colocalizes with the mitochondrial marker HSP70 (Fig. 2A). The precise localization of endogenous TgPrpB was assessed on RH wild-type parasites using specific antisera raised against the bacterially expressed recombinant TgPrpB. Endogenous TgPrpB localized to the cytosol as well as in association with the mitochondrion (Fig. 2A).

To confirm the differential distribution of the 2-MCC enzymes between the mitochondrion and cytosol of *T. gondii*, cell fractionation experiments were performed using low concentration of digitonin followed by centrifugation to separate organellar and cytosolic fractions as previously described (Pino *et al*., 2010). Consistent with the IFA results, the subcellular TgPrpE and TgPrpC are found only in the organellar fraction, whereas TgPrpD appeared exclusively in the cytosol (Fig. 2B). Endogenous aconitase (TgACN) was found predominantly in the organellar fraction but also in the cytosol (Fig. 2B) as reported for yeast (Uchiyama *et al*., 1982; Shlevin *et al*., 2007). Endogenous TgPrpB was found exclusively in the cytosolic fraction (Fig. 2C), which contrasts with the mitochondrial signal observed by IFA (Fig. 2A). This indicated that TgPrpB might not be targeted into the mitochondrion but be rather associated with the periphery of the organelle. A C-terminally Ty-tagged version of TgPrpB (PrpBTy) was also found in the cytosolic fraction whereas an N-terminally myc-tagged TgPrpB (mycPrpB) was partitioned in both the cytosol and organellar fractions (Fig. 2C). Intriguingly, mycPrpB is produced as a precursor of ∼ 40 kDa (pPrpB = precursor of PrpB) that corresponds to the predicted size of full-length PrpB and this form is detectable with anti-myc. The processed form that migrates as ∼ 36 kDa (mPrpB = mature PrpB) is only detectable with anti-PrpB antibodies (Fig. 2C). Taken together these results suggest that TgPrpB is N-terminally processed but the significance of this modification and a connection with mitochondrial targeting could not be established.

### The 2-MCC plays a dual role in detoxification and conversion of carbon sources

In bacteria and fungi, the expression of 2-MCC genes is drastically induced in the presence of the toxic compound propionic acid (Brock and Buckel, 2004; Palacios and Escalante-Semerena, 2004; Ewering *et al*., 2006). In order to establish a possible link between the presence of 2-MCC in Coccidia and a role in propionic acid detoxification, we tested if the exposure of *T. gondii* tachyzoites to increasing amounts of propionic acid would lead to a change in expression levels of one of the enzymes of the 2-MCC. Western blot analysis using the anti-PrpB revealed no such upregulation at the protein level (data not shown).

**Fig. 3:**
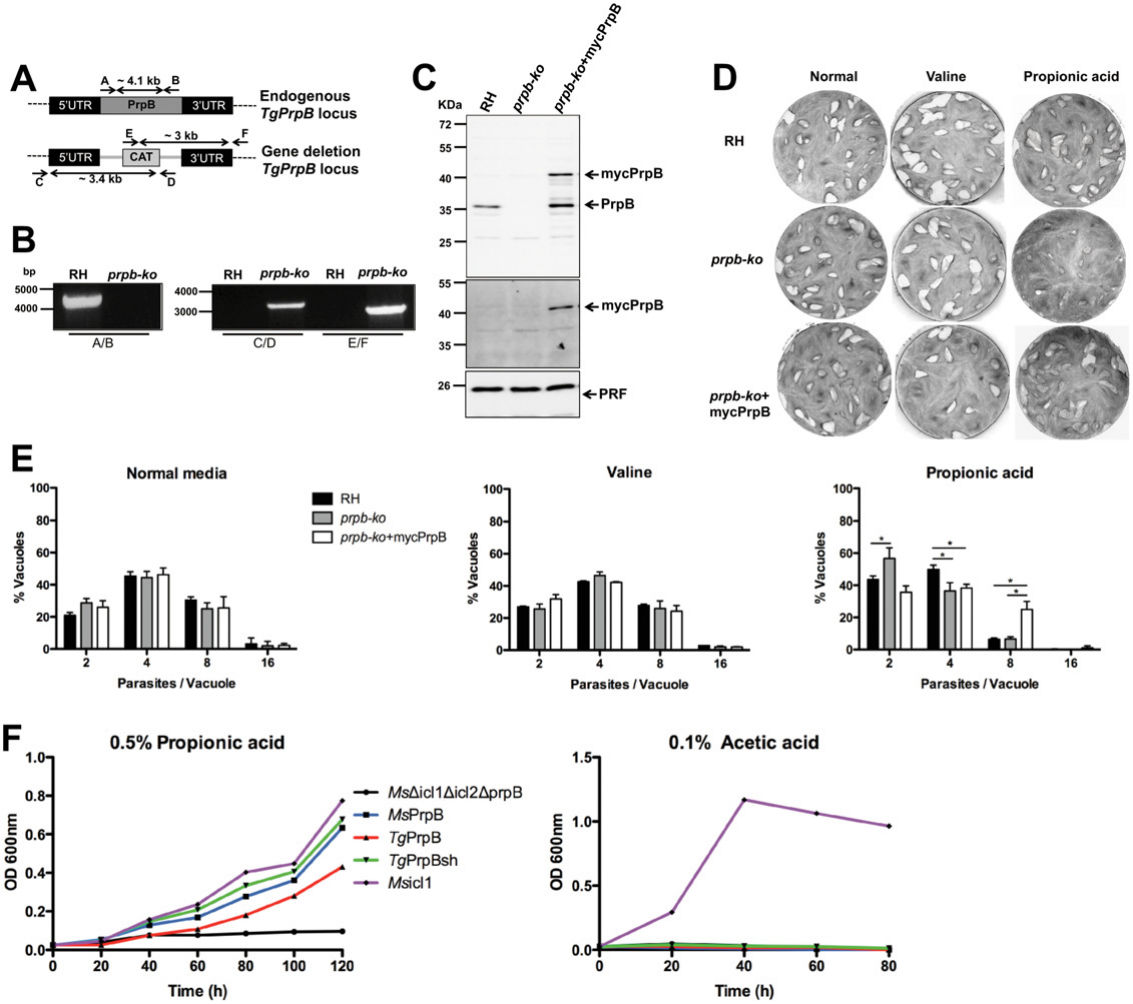
The 2-MCC contributes only modestly to the parasite growth and is implicated in detoxification of propionate and propionyl-CoA. A. Strategy used for generation of TgPrpBKO (*prpb-ko*) and the position of the primers used to confirm the knockout as well as the size of the expected amplified fragments. B. Gel shows PCR performed on genomic DNA. C. Western blot analysis using anti-TgPrpB on total extract from wild-type RH, *prpb-ko* and *prpb-ko+*mycPrpB. Anti-myc was used to detect complement tagged copy. Anti-TgPRF was used as loading control. D. Plaque assays were performed on RH, *prpb-ko* and *prpb-ko+*mycPrpB strains while exposed to different media. Parasites were grown in normal DMEM medium complemented or not with 10 mM valine or 10 mM propionic acid. E. Intracellular growth assays performed after 24 h of growth under the specified conditions (as described in D) comparing RH and *prpb-ko* as well as *prpb-ko+*mycPrpB parasites. Mean values and standard deviations are represented and reflect results from three independent biological experiments (*n* = 3) (*P < 0.05; Student *t*-test). F. Growth assays for *M. smegmatis* Δ*icl1*Δ*icl2*Δ*prpB* complemented with MsPrpB, MsIcl1, TgPrpB or TgPrpBsh respectively. Growth was tested in medium containing 0.5% propionic acid or 0.1% acetic acid as sole carbon source. The growth curve from a single experiment is shown and is representative of three independent biological experiments.

To more directly assess the importance and role of the 2-MCC for *T. gondii*, *TgPrpB* gene was disrupted by double homologous gene replacement in RH tachyzoites (Fig. 3A). Gene deletion of a *prpb-ko* clone was assessed by genomic PCR using oligonucleotide primers depicted in the scheme (Fig. 3B) and confirmed by Western blot analysis (Fig. 3C). The success in isolating parasites lacking *TgPrpB* established that the 2-MCC is dispensable for parasite growth in normal tissue culture conditions. The *prpb-ko* was complemented by expression of mycTgPrpB (Fig. 3C). In contrast TgPrpBTy is not a suitable option for complementation assays since the intact C-terminus is important for proper function of the enzyme. The published crystal atomic resolution of PrpB revealed a tetrameric structure where subunits are linked via their C-termini, suggesting that a C-terminal tag would interfere tetramer formation and activity (Grimm *et al*., 2003).

The phenotype of *prpb-ko* was first examined by plaque assay under normal growth condition and the parasite was found to form similar sized plaques as the wild-type parasites (RH) (Fig. 3D). Similarly, intracellular growth assays under the same conditions showed comparable growth between mutant and wild-type parasites (Fig. 3E). Various growth conditions were then tested to analyse the usage of alternative carbon sources. Interestingly in presence of an excess of valine (10 mM), which was expected to lead to increased levels of toxic propionyl-CoA, *prpb-ko* formed plaques of similar size as wild-type parasites (Fig. 3D) and intracellular growth was not significantly altered (Fig. 3E). In contrast, *prpb-ko* formed significantly smaller plaques than wild-type parasites in presence of propionate (10 mM) (Fig. 3D). The decrease in plaque size was due to a defect in intracellular growth (Fig. 3E) and not to a potential toxic effect on the extracellular parasite since invasion efficiency was not affect by pretreatment with 10 mM propionate (Fig. S3). The propionate-induced growth defect of *prpb-ko* was rescued when the strain was complemented by expression of mycPrpB (Figs 3D and E), confirming the role of 2-MCC in detoxifying the endogenously generated propionyl-CoA.

*Toxoplasma gondii* also possesses a mitochondrial NADP-dependent isocitrate dehydrogenase (TGME49_113140) (Pino *et al*., 2007) which could be inhibited by 2-methylisocitrate (Brock, 2005). The putative export of 2-methylcitrate to the cytosol might in itself be a detoxifying measure in this respect. This suggests a minor role of TgPrpB in parasite replication *in vitro* at least during the lytic phase of the tachyzoite life cycle. When *prpb-ko* or wild-type parasites were injected intraperitoneally into mice, both groups of animals presented signs of severe infection with the same timing. All the animals had to be sacrificed on day 6 (Table S2). The *prpb-ko* was generated in RH, a highly virulent strain that cannot form cysts, and hence infection by the oral route was not possible. In consequence, deletion of 2-MCC does not impact on virulence of RH strain; however, we cannot exclude a potential implication of this pathway in the intestine of the host following the natural route of infection.

As expected, the complementation phenotype is not recapitulated in the *prpb-ko+*PrpBTy strain when grown in the same conditions, confirming that the C-terminal tagging interferes with PrpB enzymatic activity (data not shown).

### TgPrpB complements methylisocitrate lyase deficiency in *M. smegmatis*

The biochemical activity of TgPrpB was assessed by heterologous expression in *M. smegmatis* where the gene functionally complements the PrpB activity-deficient strain of *Ms*Δ*icl1*Δ*icl2*Δ*prpB* (Upton and McKinney, 2007). Since TgPrpB carries a putative mitochondrial targeting signal, two versions of TgPrpB were used for complementation, one with and the other without the putative mitochondrial targeting sequence TgPrpB and TgPrpBsh (short) respectively (Fig. S4). When grown on propionate, *Ms*Δ*icl1*Δ*icl2*Δ*prpB* failed to grow, whereas the strain complemented with either *Ms*PrpB, *Ms*Icl1 (Upton and McKinney, 2007) or *Tg*PrpBsh sustained comparable growth (Fig. 3F). The complementation with *Tg*PrpB gave an intermediate phenotype suggesting that the N-terminal extension might interfere slightly with PrpB expression or activity in *M. smegmatis*. This result confirms that *Tg*PrpB functions as a 2-methylisocitrate lyase (MCL) (Fig. 3F). In *M. tuberculosis* the ICL enzymes can be responsible for both isocitrate lyase/2-methylisocitrate lyase ICL/MCL activities (Munoz-Elias *et al*., 2006). To determine if *Tg*PrpB functions as a MCL only or as an ICL/MCL, the *M. smegmatis* complemented strains were grown on acetate as sole carbon source. The parent strain (*Ms*Δ*icl1*Δ*icl2*Δ*prpB*) is devoid of ICL activity and therefore cannot grow on acetate. As expected the strain complemented with the *Ms*Icl1 restored growth of the parental strain on acetate. None of the other strains complemented with TgPrpB were able to restore growth on acetate indicating that TgPrpB functions solely as a MCL (Fig. 3F).

### Accumulation of 2-MCC intermediate metabolites in *prpb-ko* mutant

**Fig. 4:**
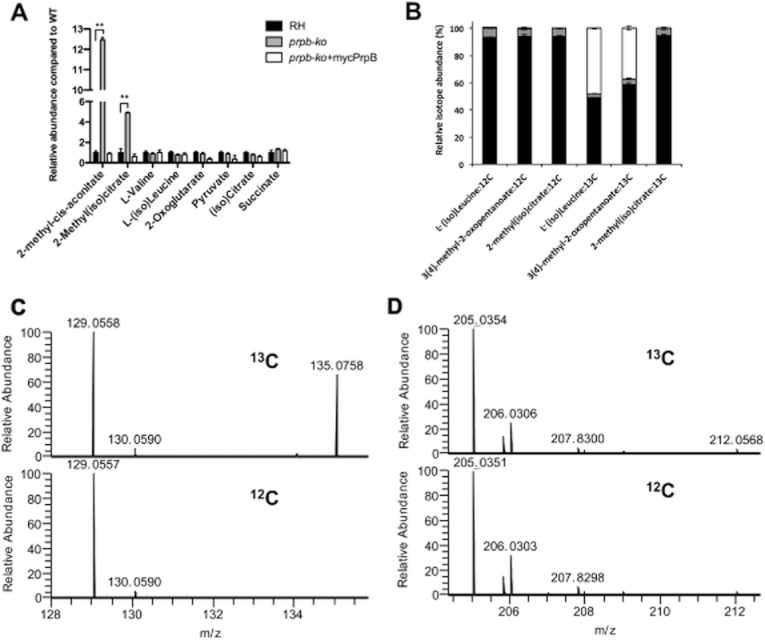
Labelled branched-chain amino acids are converted to the corresponding branched-chain ketoacids, but not into 2-methylcitrate. A. Histogram represents mean relative metabolite abundances from *prp-bko* or *prpb-ko+*mycPrpB vs. RH parasites. Statistics (Student *t*-test) and standard deviation reflect three independent biological replicates (*n* = 3) (**P < 0.01; Student *t*-test). 2-Methyl-cis-aconitate is putatively identified based on exact mass, others identified by exact mass and retention time with authentic standards, single (overlapping) peak measurement shown for 2-methylcitrate/2-methylisocitrate isomers and for l-leucine/l-isoleucine isomers. B. Unlabelled (filled columns) and labelled (open columns) metabolite abundances from wild-type *T. gondii* tachyzoites grown in the presence of 0.8 mM uniformly labelled (^13^C) and 0.8 mM unlabelled (^12^C) or 1.6 mM of unlabelled (^12^C) leucine, isoleucine and valine. Grey columns represent the natural isotope abundances of carbon-13. Error bars represent the standard deviation of three independent biological replicates (*n* = 3). Each metabolite likely represents the combination of two isomeric metabolites as indicated in parentheses. C. Example mass spectrum of 3(4)-methyl-2-oxopentanoate (*m/z* = 129.056; RT = 4.5 min) after incubation with uniformly labelled (^13^C) or unlabelled (^12^C) branched-chain amino acids. The peak at 135.076 represents the six-carbon labelled metabolite derived from l-(iso)leucine. D. Example mass spectrum of 2-methyl(iso)citrate (*m/z* = 205.035; RT = 18.7 min) after incubation with labelled (^13^C) or unlabelled (^12^C) branched-chain amino acids. The absence of peaks at 208.045 precludes incorporation of three carbons from branched-chain amino acid-derived propanoate.

We further investigated the metabolic profile of extracellular tachyzoites using liquid chromatography-mass spectrometry (LC-MS). The *prpb-ko* strain presents higher amounts of 2-methylcitrate/2-methylisocitrate (two isomers undistinguishable by LC-MS) and 2-methyl-cis-aconitate (putative), compared to the wild-type strain, while other TCA cycle intermediates including citrate and succinate, pyruvate and amino acids were present in similar amounts to the wild-type. Accumulation of the 2-MCC intermediates was abolished upon complementation of *prpb-ko* with mycPrpB, confirming the enzymatic activity of TgPrpB and the specificity of the block occurring in absence of TgPrpB (Figs 4A and S5A–H, Table S3).

To understand how the propionyl-CoA could be generated in the parasite, we undertook metabolic profiling experiments combined with ^13^C labelling. Extracellular wild-type parasites were incubated in presence of 50% U-^13^C-labelled or 100% ^12^C BCAA mix (valine, leucine and isoleucine) and incorporation of the ^13^C carbons in the different metabolites was assessed by LC-MS. As expected, we detected a 1:1 ratio of labelled (white column) : unlabelled (black column) in the l-(iso)leucine control in the presence of ^13^C BCAA and not in the presence of ^12^C BCAA, which was fully unlabelled (Fig. 4B). In addition, we observed incorporation of ^13^C carbons into the 3(4)-methyl-2-oxopentanoate ketoacid generated from the deamination of l-(iso)leucine by the branched-chain aminotransferase (BCAT) (Figs 4B and C). Interestingly, we observed no incorporation of ^13^C carbons into the 2-methyl(iso)citrate, indicating that the BCAA degradation pathway is not fully functional in *in vitro* culturing conditions and probably not the source of propionyl-CoA inducing the accumulation of 2-methyl(iso)citrate in the *prpb-ko* that we observe (Figs 4B and D). As *T. gondii* possesses all the enzymes implicated in β-oxidation of fatty acids except for the carnitine/acylcarnitine carrier, we cannot exclude propionyl-CoA being generated through the degradation of odd-chain fatty acids as being the source of propionyl-CoA for the 2-MCC.

Taken together these results indicate that *Tg*PrpB functions as a 2-methylisocitrate lyase in *T. gondii* and belongs to the pathway responsible for the detoxification of propionic acid.

### Branched-chain amino acid degradation is dispensable in *T. gondii*

**Fig. 5:**
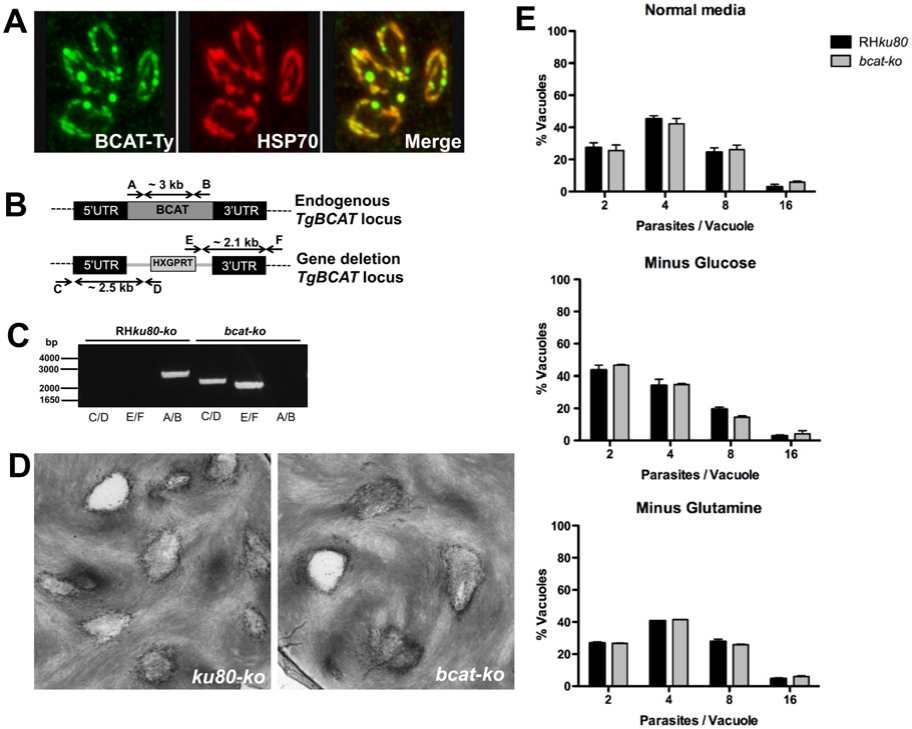
Branched-chain amino acid degradation is fully dispensible in *T. gondii*. A. Localization of the *T. gondii* branched-chain aminotransferase (TgBCAT) was assessed by IFA on tachyzoites expressing a C-terminally Ty-tagged copy of BCAT. IFA shows colocalization of the protein (in green) with the mitochondrial marker TgHSP70 (in red). B. Strategy used for generation of *TgBCAT* (*bcat-ko*) and the position of the primers used to confirm the knockout as well as the size of the expected amplified fragments. C. PCR performed on genomic DNA using primers described in A confirms gene deletion. D. Plaque essay performed on RH*ku80-ko* (wild type) or *bcat-ko*. E. Intracellular growth assay was performed in presence of different carbon sources. Upper panel shows growth in complete media, middle panel shows growth in media lacking glucose and lower panel shows growth in media lacking glutamine. The data shown are the means and standard deviations of three independent experiments (*n* = 3).

The absence of toxicity when *prpb-ko* was cultivated in presence of excess of valine (Figs 3D and E) and the lack of incorporation of ^13^C-labelled BCAA (Fig. 4B) into the 2-MCC questioned the importance of the branched-chain amino acid degradation pathway for survival of *T. gondii* tachyzoites. To address this issue directly, we characterized the first enzyme of the pathway TgBCAT. A stable parasite line expressing a C-terminal epitope-tagged version of the protein was generated (*Tg*BCAT-Ty) and shown to colocalize with TgHSP70 by IFA (Fig. 5A). *TgBCAT* was then disrupted by double homologous recombination and a *bcat-ko* clone was assessed by genomic PCR using primers depicted on the scheme (Fig. 5B). When assessed by plaque assay, the *bcat-ko* formed plaques of similar size as wild-type parasites further supporting the dispensability of BCAA degradation in this parasitic stage (Fig. 5C). In addition, *bcat-ko* parasites exhibited no obvious defect in intracellular growth assay (Fig. 5D). To address the importance of different carbon sources in absence of BCAA degradation, we assessed parasitic growth in media lacking glucose or glutamine but could not observe any growth differences between a *bcat-ko* and wild-type parasites (Fig. 5D) indicating that BCAA degradation is fully dispensable for tachyzoite propagation in tissue culture conditions.

## Discussion

In most eukaryotes, mitochondrial metabolism responds to anabolic needs; however, the metabolic contribution of the single tubular mitochondrion has remained elusive in *T. gondii* and other apicomplexans (Seeber *et al*., 2008). Indeed, the exclusive localization of the PDH complex to the relict plastid (apicoplast) (Foth *et al*., 2005; Fleige *et al*., 2007) has been puzzling, as this requires the TCA cycle to function without the mitochondrial conversion of glucose-derived pyruvate into acetyl-CoA (Ralph, 2005). A recent study by Macrae *et al*. shows that *T. gondii* does possess a canonical oxidative TCA cycle that is fuelled by glucose through its conversion to acetyl-CoA although how the acetyl-CoA is generated is still unknown (Macrae *et al*., 2012).

Bioinformatics searches aiming at the identification of pathways able to provide a sustainable acetyl-CoA source in the mitochondrion were inconclusive regarding fatty acid beta-oxidation, but indicated the presence of the genes coding for the BCAA pathway uniquely in Coccidia (Seeber *et al*., 2008). While both pathways could ultimately generate acetyl-CoA within the mitochondrion, the concomitant production of potentially cytotoxic propionyl-CoA would require a detoxification mechanism.

The complete set of enzymes implicated in the 2-MCC has been preserved in the coccidian branch of Apicomplexa. The dinoflagellates belong to the group of Alveolata but are free-living species relying on photosynthesis and have also retained the 2-MCC (Danne *et al*., 2012). Interestingly, in *T. gondii*, these enzymes are distributed between the mitochondrion and the cytosol. The first two steps of propionic acid detoxification are performed by TgPrpE and TgPrpC and take place inside the mitochondrion. In this context, the fungal PrpB activity was previously detected in both cytosol and mitochondria (Uchiyama *et al*., 1982). In these species, the 2-MCC likely starts in the mitochondrion and relocates to the cytosol after the generation of 2-methylcitrate by PrpC. It has been proposed that this metabolite could be exported via the tricarboxylic acid transporter in the inner membrane of the mitochondrion (Cheema-Dhadli *et al*., 1975). Such a putative transporter has been identified in the *T. gondii* genome and harbours a mitochondrial targeting signal (Table 1). The next enzyme of the pathway, TgPrpD, is clearly cytosolic. The aconitase is more complex since TgACN was described earlier as a dually targeted mitochondrial and apicoplast protein (Pino *et al*., 2007). Here we used anti-TgACN antibodies to avoid artefact of overexpression and detected TgACN both in the organellar (in majority) and in the cytosolic factions. TgPrpB is also cytosolic despite an N-terminal cleavage and a partial association with the mitochondrion. TgPrpB is implicated in the last step of the 2-MCC and produces pyruvate and succinate that can be used for different reactions, both inside and outside of the mitochondrion. Both end products of the 2-MCC can enter the mitochondrion via the mitochondrial dicarboxylic and monocarboxylic acid transporters, respectively, to merge with the TCA cycle. Disruption of TgPrpB revealed the non-essentiality of the 2-MCC pathway in parasites grown in rich media. In the plant pathogenic fungi Gibberellazeae, an isocitrate lyase (ICL) compensates for the lack of PrpB (Lee *et al*., 2009). In *T. gondii*, the ICL enzyme is absent, thus precluding such compensation. Studying the intracellular growth of the parasites revealed that the strain overexpressing TgPrpB is able to rescue the growth defect of *prpb-ko* when exposed to exogenous propionate leading to accumulation of endogenously produced toxic compounds such as propionate-derived molecules, propionyl-CoA and 2-methylcitrate. A previous study performed on methylisocitrate lyases revealed, in accordance with our results, that TgPrpB is most closely related to bacterial methylisocitrate lyases with a sequence identity of up to 50% (Muller *et al*., 2011). TgPrpB was considered a putative methylisocitrate lyase because of its exotic active site and no further data provided evidence of its enzymatic activity (Muller *et al*., 2011). 2-Methylisocitrate is not commercially available making it difficult to directly assess PrpB's enzymatic activity. However, the sensitivity of *prpb-ko* to propionic acid together with the efficient complementation of TgPrpB for *M. smegmatis* grown on propionate supports a role for the 2-MCC in detoxification and no significant role of carbon conversion in an anaplerotic fashion as described in mycobacteria (Upton and McKinney, 2007). In addition the metabolomic analysis carried out in this study further confirms the 2-methylisocitrate lyase activity of PrpB in *T. gondii* as found in the heterologous complementation performed in *M. smegmatis*. The metabolomic analysis combined with ^13^C labelling also highlighted the absence of branched-chain amino acid degradation in *in vitro* tachyzoites, indicating that the propionyl-CoA fuelling the 2-MCC in this condition must be generated elsewhere, possibly by the β-oxidation of odd fatty acids. This was further supported by the non-essentiality of *TgBCAT* and the absence of phenotype in the tachyzoites.

Given the common enteric host niche they occupy, the Coccidia are exposed to high levels of propionate in the host digestive tracts and potentially to endogenously produced propionyl-CoA as a product of BCAA. These metabolites exert inhibitory effects on other CoA-dependent enzymes, such as BCKDH, but also on the succinyl-CoA synthetase and the ATP citrate lyase, all of which are present in coccidian parasites (Seeber *et al*., 2008) justifying the retention of the 2-MCC for detoxification purposes. Finally *T. gondii* undergoes sexual reproduction in the gut of felids and we cannot rule out a significant role of the 2-MCC for survival and development in the intestine of the definitive host.

## Experimental procedures

### Bioinformatic and phylogenetic analysis

Preliminary genome data were accessed from the *Toxoplasma* Genome Sequencing Consortium (http://ToxoDB.org) and from the US Department of Energy, DOE Joint Genome Institute (http://genome.jgi-psf.org/ramorum1/ramorum1.home.html). Genomic data were provided by the Institute for Genomic Research (NIH Grant #AI05093), and by the Wellcome Trust Sanger Institute. EST sequences were generated by Washington University (NIH Grant #1R01AI045806-01A1). Sequence data of *Phytophthora* spp. were produced by the US Department of Energy Joint Genome Institute (http://www.jgi.doe.gov/) (project leaders: Jeffrey Boore and Daniel Rokhsar) in collaboration with the Virginia Bioinformatics Institute (http://phytophthora.vbi.vt.edu) (project leader: Brett Tyler). Protein sequences related to MCC enzymes were identified by BLAST searching, and additional sequences were downloaded from GenBank (accession numbers are given in the figures and in supplementary text files PrpB.txt and PrpD.txt). Before generating the alignments for phylogenetic analysis we identified (by inspecting alignments and by extensive pairwise BLASTing) and deleted insertions that were present in very few or individual sequences. Multiple protein sequence alignments were generated with ProbCons v1.12 (Do *et al*., 2005) at http://www.phylogeny.fr (Dereeper *et al*., 2008) using default parameters (consistency reps = 2, iterative refinement reps = 100, pre-training reps = 0), and alignment positions containing gaps in more than 50% of the sequences were excluded from phylogenetic analyses. Maximum-likelihood trees were calculated using PhyML v2.4.5 (Guindon and Gascuel, 2003) with the following parameters: JTT amino acid substitution model with an estimated proportion of invariable sites and gamma distribution parameter, with four substitution rate categories, a BIONJ starting tree and optimized tree topology and optimized branch lengths and rate parameters. Distance matrix-based phylogenetic analyses were carried out using the neighbour-joining algorithm by employing the PHYLIP (v.3.69) programs ProtDist, Neighbor, SeqBoot and Consense (Felsenstein, 2005). The Jones–Taylor–Thornton (JTT) amino acid substitution matrix was used in ProtDist, and input order of species for phylogenetic analysis was always randomized. Bootstrap values were calculated based on 100 pseudoreplicates, and phylogenetic trees were visualized using TreeView. Mitochondrial import signals were predicted *in silico* using TargetP 1.1- and MitoprotII- algorithms (Claros and Vincens, 1996; Emanuelsson *et al*., 2007).

### Bacteria, parasite strains and culture

*Escherichia coli* XL-10 Gold and BL21 (DE3) were used for recombinant DNA and protein expression respectively. *T. gondii* tachyzoites were grown in human foreskin fibroblasts (HFF) or in Vero cells (African green monkey kidney cells) maintained in Dulbecco's modified Eagle's medium (DMEM; Gibco, Invitrogen) supplemented with 5% fetal calf serum (FCS), 2 mM glutamine and 25 μg ml^−1^ gentamicin.

### Cloning of *T. gondii* genes for 2-MCC

*TgPrpC*, *TgPrpD*, *TgPrpB*, N-terminal fragment of *TgPrpE* (first 280 amino acid of the protein) and TgBCAT cDNA were amplified by PCR from *T. gondii* RH total cDNA, initially ligated into the commercial pGEM T-easy vector (Promega), and digested with appropriate restriction enzymes (New England Biolabs). Primers and restriction enzymes are listed in Table S1. For recombinant protein expression, *TgPrpB* cDNA was ligated into pET3b modified with a stretch of six histidine residues at the C-terminus (Invitrogen). For expression in the parasite, plasmids pTgPrpC-Ty, pTgPrpD-Ty, pTgPrpB-Ty and pTgBCAT-Ty were generated by cloning the respective cDNA between the EcoRI and NsiI or SbfI sites in the pT8MLCTy-HX vector (Herm-Gotz *et al*., 2002) or the pT8-GFP-TY-HX for PrpE and between the NsiI and PacI sites in the pT8MycGFPMyoAtail-HX vector for the pmyc-TgPrpB plasmid. Ty1 tag sequence has been previously reported (Bastin *et al*., 1996).

Two kilobases of the 5′ and 3′ flanking regions of TgPrpB's ORF were cloned into the pTub5CAT. Plasmid was then transfected in wild-type RH to create the *prpb-ko* transgenic line as described in the following section.

Two kilobases of the 5′ and 3′ flanking regions of *TgBCAT* were cloned into the pTub5HXGPRT knockout vector. Plasmid was transfected in the wild-type RH*ku80-ko* to create the transgenic *bcat-ko* transgenic line. Primers for the PCRs and used restriction enzymes sites are listed in Table S1.

### Generation of parasite transgenic lines

*Toxoplasma gondii* tachyzoites [RH wild-type or RH *ku80-ko* (Huynh and Carruthers, 2009)] were transfected by electroporation as previously described (Soldati and Boothroyd, 1993). Stable transfectants were selected for by hypoxanthine-xanthine-guanine-phosphoribosyltransferase (HXGPRT) expression in the presence of mycophenolic acid and xanthine as described earlier (Donald *et al*., 1996) or by resistance to 20 μg ml^−1^ chloramphenicol added on the day of transfection in the case of *TgPrpB*. Parasites were cloned by limiting dilution in 96-well plates and analysed for the expression of the transgenes by indirect immunofluorescence assay (IFA) for expression of epitope-tagged proteins or by genomic PCR and Western immunoblotting for the *prpb-ko* and by PCR for the *bcat-ko* mutants.

### Generation of polyclonal antisera against *T. gondii* PrpB and TgACN

Part of the *TgPrpB* gene was amplified using primers 1428 and 1363 (Table S1). The 1100 bp fragment was cloned between the EcoRI and the SbfI sites into the pTrcHisTOPO vector. Expression of recombinant protein was induced with 1 mM isopropyl-beta-d-thiogalactopyranoside (IPTG). The hexahistidine fusion proteins were purified using a NiNTAagarose column, under denaturing conditions according to the manufacturer's instructions.

Two peptides from TgACN were used for antibody production. The first corresponding to amino acids 526 to 541 (PCVAGPKRPQDRVPLS) and the second corresponding to amino acids 858 to 872 (KGVERKDFNTYGARR).

Antibodies against TgPrpB and TgACN were raised in rabbits by Eurogentec S.A. (Seraing, Belgium) according to their standard protocol.

### Indirect immunofluorescence confocal microscopy

Intracellular parasites grown on HFF coverslips were washed with PBS and fixed after 24 h with 4% paraformaldehyde for 10 min and neutralized with PBS containing 0.1 M glycine. Cells were then permeabilized in PBS, 0.2% Triton-X-100 for 20 min and blocked in the same buffer with 2% BSA. Slides were incubated for 1 h with primary antibodies [mouse monoclonal anti-Ty (BB2), mouse monoclonal anti-myc (9E10), rabbit anti-MLC1, rabbit anti-TgGAP45 (Plattner *et al*., 2008), rabbit anti-TgHSP70 (Pino *et al*., 2010) and mouse anti-TgActin], washed and incubated for 1 h with Alexa 594 goat anti-rabbit or Alexa 488 goat anti-mouse antibodies (Molecular Probes). Finally coverslips were incubated 10 min with 4′,6-diamidino-2-phenylindole (DAPI) in PBS, and mounted in FluoromountG (Southern Biotech). Confocal images were collected with a Leica laser scanning confocal microscope (TCS-NT DM/IRB) using a 63× Plan-Apo objective with NA 1.40. Single optical sections were recorded with an optimal pinhole of 1.0 (according to Leica instructions) and 16 times averaging.

### Western blot analysis

Crude extracts of *T. gondii* tachyzoites were subjected to SDS-PAGE as described previously (Soldati *et al*., 1998). Western blot analysis was carried out using 12% polyacrylamide gels run under reducing conditions. Proteins were transferred to hybond ECL nitrocellulose. For detection, the membranes were incubated with primary antibodies [rabbit anti-TgPrpB, mouse monoclonal anti-Ty (BB2), mouse monoclonal anti-myc (9E10), rabbit anti-TgProfilin (PRF), rabbit anti-Aconitase (ACN), rabbit anti-Cpn60 (Agrawal *et al*., 2009)] diluted in PBS, 0.05% Tween20, 5% skimmed milk. Secondary antibodies used were horseradish peroxidase-conjugated goat anti-mouse IgG or goat anti-rabbit IgG (Bio-Rad). Bound antibodies were visualized using the ECL system (Amersham).

### Subcellular fractionation of *T. gondii*

Parasites were washed in PBS and resuspended in SoTE buffer (0.6 M sorbitol, 20 mM Tris-HCL, pH 7.5, and 2 mM EDTA). Samples were mixed with SoTE containing a final concentration of 0.1%, 0.05% or 0.025% of digitonin. Incubation was allowed to proceed on ice for 5 min. Treated cells were then centrifuged (800 *g*, 5 min, 4°C) and supernatant corresponding to the cytosolic fraction was separated from the pellet corresponding to the organellar fraction. Both fractions were resuspended in protein loading buffer and used for Western blots analysis. Organellar integrity was assessed using anti-HSP70 (mitochondria) (Pino *et al*., 2010) and anti-Cpn60 (apicoplast) (Agrawal *et al*., 2009).

### Phenotypic analysis

Prior to infection, the HFF monolayers were washed with the DMEM medium containing the desired carbon source and kept in this medium for the rest of the experiment.

Plaque assays: HFF were grown to form monolayers on coverslips. Cells were infected with parasites and let to develop for 7 days. Fixation and staining were performed as described in Plattner *et al*. (2008).

Intracellular growth assays: similarly to plaque assays HFF were infected with parasites in presence of different carbon sources. When indicated, 10 mM valine or propionic acid was added. Where indicated, freshly egressed parasites were incubated for 45 min in media containing ± 10 mM propionic acid prior infecting the HFF monolayer. After 24 h, IFAs were performed using anti-TgGAP45 and the number of parasites per vacuole was counted. Two hundred vacuoles were counted for each condition. The data shown are the means and standard deviations from three independent experiments.

### Invasion assay red/green

Freshly egressed parasites were incubated for 45 min in low-glucose media ± 10 mM propionic acid prior the invasion assay. Parasites were then allowed to invade a new host cell layer for 45 min before being fixed with PAF/Glu for 7 min. Cells were neutralized 3–5 min in 0.1 M glycine/PBS and blocked 20 min with 2% BSA/PBS (no permeabilization). Cells were then incubated with mouse anti-SAG1 antibodies diluted in 2% BSA/PBS for 20 min and washed three times with PBS. The cell layer was then fixed again with 1% formaldehyde/PBS for 7 min and washed once with PBS. An IFA was then performed as described earlier using anti-TgGAP45 antibodies. Two hundred parasites were counted for each strain and the percentage of intracellular parasites is represented. The data shown are the means and standard deviations from three independent experiments.

### *In vivo* virulence assay

On day 0, mice were infected by intraperitoneal injection with either wild-type RH or *prpb-ko* parasites (∼ 30–50 parasites per mouse). Five mice were infected per group. The health of the mice was followed daily until they presented severe symptoms of acute toxoplasmosis (bristled hair and complete prostration with incapacity to drink or eat) and were sacrificed on that day in accordance to the Swiss regulations of animal welfare.

### *Mycobacterium smegmatis* complementation and growth assays

The plasmids pMV261-TgPrpB, pMV261-TgPrpBsh and pMV261-MsIcl1 were constructed by cloning the PCR-amplified *T. gondii prpB* ORF [full length or without the N-terminal (sh)] or the *M. smegmatis icl1* ORF into the vector pMV261 (Upton and McKinney, 2007). PCR was performed to amplify the respective amplicons, with primers providing 5′ and 3′ HindIII sites. The plasmids, including the positive controls pPRPB (Upton and McKinney, 2007) and pMV261-Icl1, were electroporated into *M. smegmatis* and positive transformants were selected on 7H10 agar containing kanamycin (20 μg ml^−1^).

The above bacterial strains were grown at 37°C with aeration in Middlebrook 7H9 broth (DIFCO) supplemented with 0.5% BSA, 0.085% NaCl, 0.05% Tween-80 and 0.2% glucose (Sigma). For assessment of carbon utilization, bacteria were grown at 37°C in M9 broth [composed of M9 salts (DIFCO), supplemented with 0.1 mM CaCl_2_, 2 mM MgSO_4_ (Sigma)] containing 0.5% (w/v) propionic acid or 0.1% (w/v) acetic acid. Bacteria were cultivated using a shaking platform for 120 h, with samples taken at 20 h intervals for growth assessment using OD600 measurements.

### Metabolic profiling

5 × 10^8^ freshly egressed *T. gondii* tachyzoites cultivated in complete media at 37°C in 5% CO_2_ were transferred to prechilled Falcon tubes and immediately quenched to 0°C in an ethanol-dry ice bath to stop metabolic reactions. For ^13^C labelling experiment, extracellular tachyzoites were incubated for 4 h in media containing 50/50 ratio of ^13^C/^12^C (labelled) or 100% ^12^C (unlabelled) BCAA mix (valine, leucine, isoleucine from CIL, Researchem Lifescience). Metabolites were then extracted as following. Extracellular parasites were centrifuged at 1000 *g* for 10 min and all supernatant was discarded. Two hundred microlitres of cold (0°C) chloroform/methanol/water (1/3/1 v/v/v) was added to the pellet. Metabolite extraction was performed at 4°C for 1 h in a Thermomixer (1400 r.p.m.). After centrifugation at 10 000 *g* for 5 min at 4°C, the supernatant was transferred to a new tube, subjected to deoxygenation with a gentle stream of nitrogen gas prior to tube closure. Samples were stored at −70°C until analysis. Protocol was adapted from t'Kindt *et al*. (2010).

LC-MS analysis utilized high-resolution mass spectrometry coupled to hydrophilic interaction chromatography according to the ZIC–pHILIC ammonium carbonate method previously described (Zhang *et al*., 2012). Minor adjustments to the published method were the m/z range 70–1400, sheath gas 40, auxiliary gas 20, source voltage +4 kV and −3.5 kV, and ESI probe temperature 150°C.

All samples were analysed in the same analytical batch in randomized order and the quality of chromatography and signal reproducibility were checked by analysis of each metabolite in pooled quality control samples and visual inspection of total ion chromatograms. Three biological replicates were analysed.

Metabolite identities were confirmed by exact mass and retention time for metabolites where authentic standards were available for analysis. Putative identification of other metabolites was made on the basis of exact mass and predicted retention time (Creek *et al*., #b^12^b^13^). Quantification is based on raw peak heights, and expressed relative to the average peak height observed in wild-type cells.
