# Supplementary material for: The 2-methylcitrate cycle is implicated in the detoxification of propionate in Toxoplasma gondii
Source: Mol Microbiol. 2013 Jan 11;87(4):894–908. doi: 10.1111/mmi.12139 (PMC3593168; doi:10.1111/mmi.12139)
Supplement: Supplementary file 1 [file mmi0087-0894-SD1.zip › mmi_12139_sm_fS1-5, tS1-3.pdf]

SUPPLEMENTARY INFORMATION: The 2-methylcitrate cycle is implicated in the detoxification of propionate in *Toxoplasma gondii**

SUPPLEMENTARY FIGURE S1 An unrooted PhyML Maximum-Likelihood (ML) phylogeny (log-likelihood = -30087.002, gamma shape parameter = 1.230) of PrpB protein sequences. Bootstrap support values of at least 50% are shown above (ML) and below (Neighbor-Joining) the branches (100 pseudoreplicates). Sequences from eukaryotic organisms are represented in blue and green, whereas black represents bacterial sequences. Red designates proteins whose MCC-specific enzymatic function had previously been demonstrated biochemically. Sequence accession numbers are provided next to the species names. Sequences used to generate this phylogeny are provided in supplementary file Alignment PrpB.txt

SUPPLEMENTARY FIGURE S2 An unrooted PhyML Maximum-Likelihood (ML) phylogeny (log-likelihood = -38958.410, gamma shape parameter = 1.465) of PrpD protein sequences. Bootstrap support values of at least 50% are shown above (ML) and below (Neighbor-Joining) the branches (100 pseudoreplicates). Sequences from eukaryotic organisms are represented in blue and green, whereas black represents bacterial sequences. Red designates proteins whose MCC-specific enzymatic function had previously been demonstrated biochemically. Sequence accession numbers are provided next to the species names. Sequences used to generate this phylogeny are provided in supplementary file Alignment PrpD.txt

SUPPLEMENTARY FIGURE S3 Exogenous propionate does not affect viability of *prpb-ko* A. Wild type*, prpb-ko* or *prb-ko*+mycPrpB extracellular parasites were incubated for 45 minutes in media containing or not 10 mM propionic acid prior a red/green invasion assay to assess toxicity of exogenous propionic acid on the parasite. Mean value and standard deviation are represented and reflect results from three independent experiments (n=3). B. The same pre-treated parasites as in A were used for a 24h intracellular growth assay and shows the same growth defect of *prpb-ko* as in not pre-treated parasites (Figure 3E). Mean value and standard deviation are represented and reflect results from three independent experiments (n=3). (*< p=0.05, **< p=0.01; Student T-test).

SUPPLEMENTARY FIGURE S4 Protein alignment of *M.smegmatis* and *T.gondii* PrpBs. Arrow indicates where TgPrpB potential mitochondrial signal peptide was truncated to produce the TgPrpB short (TgPrpBsh)

SUPPLEMENTARY FIGURE S5 LC-MS spectra for various metabolites analysed. A. 2-methyl-cis-aconitate. B. 2-methyl-(iso)citrate. C. (iso)citrate. D. Succinate. E. 2-oxoglutarate. F. Pyruvate. G. Valine. H. L-(iso)leucine

SUPPLEMENTARY TABLE S1 List of primers used for this study

SUPPLEMENTARY TABLE S2 Virulence assay in mice infected with wild type or *prpb-ko* parasites.

SUPPLEMENTARY TABLE S3 LC-MS Raw peak heights for *prpb-ko* vs. wild type

Figure S1

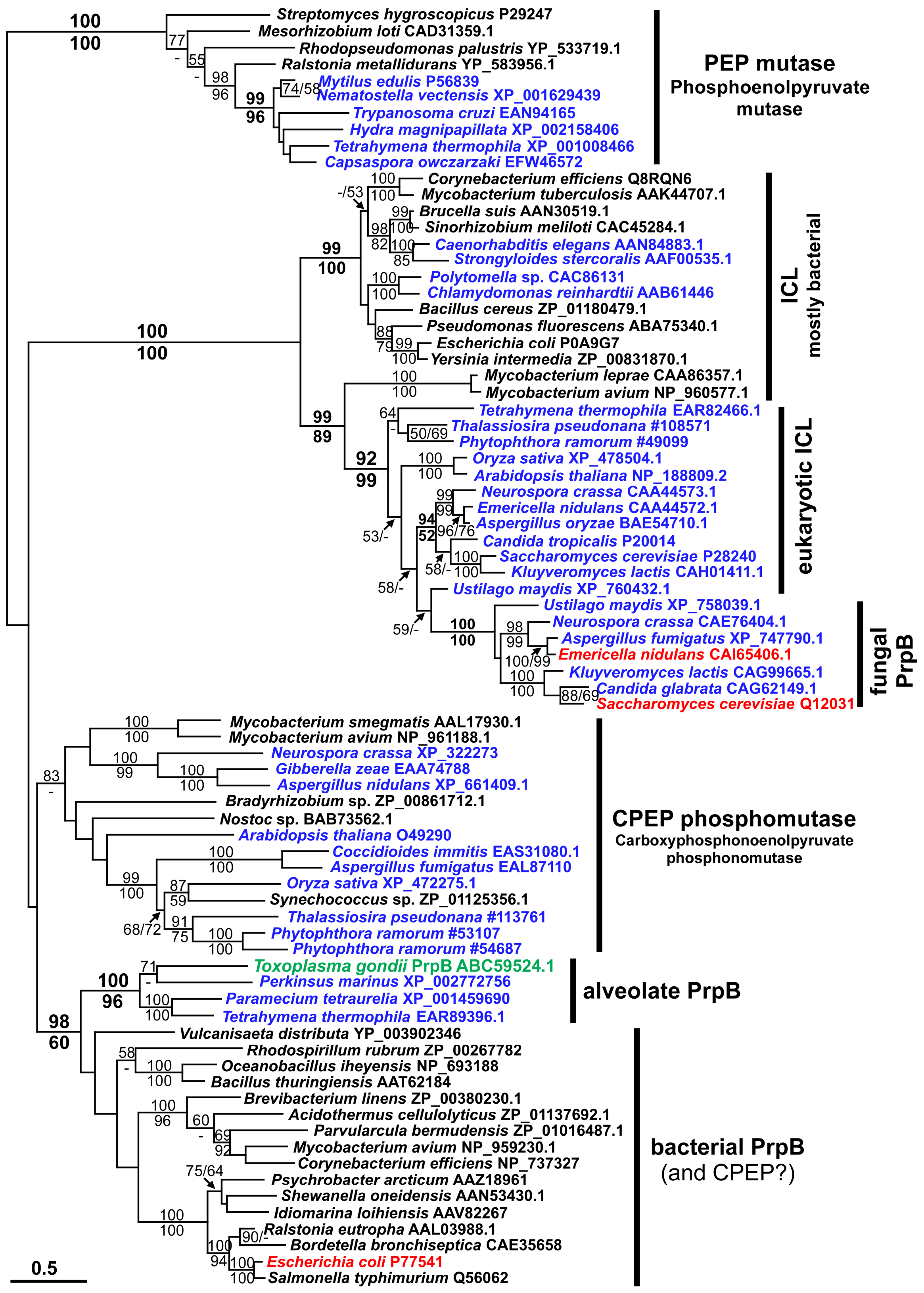

Streptomyces hygroscopicus P29247
Mesorhizobium loti CAD31359.1
Rhodopseudomonas palustris YP_533719.1
Ralstonia metallidurans YP_583956.1
Mytilus edulis P56839
Nematostella vectensis XP_001629439
Trypanosoma cruzi EAN94165
Hydra magnipapillata XP_002158406
Tetrahymena thermophila XP_001008466
Capsaspora owczarzaki EFW46572
PEP mutase
Phosphoenolpyruvate mutase
Corynebacterium efficiens Q8RQN6
Mycobacterium tuberculosis AAK44707.1
Brucella suis AAN30519.1
Sinorhizobium meliloti CAC45284.1
Caenorhabditis elegans AAN84883.1
Strongyloides stercoralis AAF00535.1
Polytomella sp. CAC86131
Chlamydomonas reinhardtii AAB61446
Bacillus cereus ZP_01180479.1
Pseudomonas fluorescens ABA75340.1
Escherichia coli P0A9G7
Yersinia intermedia ZP_00831870.1
Mycobacterium leprae CAA86357.1
Mycobacterium avium NP_960577.1
ICL
mostly bacterial
Tetrahymena thermophila EAR82466.1
Thalassiosira pseudonana #108571
Phytophthora ramorum #49099
Oryza sativa XP_478504.1
Arabidopsis thaliana NP_188809.2
Neurospora crassa CAA44573.1
Emericella nidulans CAA44572.1
Aspergillus oryzae BAE54710.1
Candida tropicalis P20014
Saccharomyces cerevisiae P28240
Kluyveromyces lactis CAH01411.1
eukaryotic ICL
Ustilago maydis XP_760432.1
Ustilago maydis XP_758039.1
Neurospora crassa CAE76404.1
Aspergillus fumigatus XP_747790.1
Emericella nidulans CAI65406.1
Kluyveromyces lactis CAG99665.1
Candida glabrata CAG62149.1
Saccharomyces cerevisiae Q12031
fungal PrpB
Mycobacterium smegmatis AAL17930.1
Mycobacterium avium NP_961188.1
Neurospora crassa XP_322273
Gibberella zeae EAA74788
Aspergillus nidulans XP_661409.1
Bradyrhizobium sp. ZP_00861712.1
Nostoc sp. BAB73562.1
Arabidopsis thaliana O49290
Coccidioides immitis EAS31080.1
Aspergillus fumigatus EAL87110
Oryza sativa XP_472275.1
Synechococcus sp. ZP_01125356.1
Thalassiosira pseudonana #113761
Phytophthora ramorum #53107
Phytophthora ramorum #54687
CPEP phosphomutase
Carboxyphosphonoenolpyruvate phosphonomutase
Toxoplasma gondii PrpB ABC59524.1
Perkinsus marinus XP_002772756
Paramecium tetraurelia XP_001459690
Tetrahymena thermophila EAR89396.1
alveolate PrpB
Vulcanisaeta distributa YP_003902346
Rhodospirillum rubrum ZP_00267782
Oceanobacillus iheyensis NP_693188
Bacillus thuringiensis AAT62184
Brevibacterium linens ZP_00380230.1
Acidothermus cellulolyticus ZP_01137692.1
Parvularcula bermudensis ZP_01016487.1
Mycobacterium avium NP_959230.1
Corynebacterium efficiens NP_737327
Psychrobacter arcticum AAZ18961
Shewanella oneidensis AAN53430.1
Idiomarina loihiensis AAV82267
Ralstonia eutropha AAL03988.1
Bordetella bronchiseptica CAE35658
Escherichia coli P77541
Salmonella typhimurium Q56062
bacterial PrpB
(and CPEP?)
0.5

Figure S2

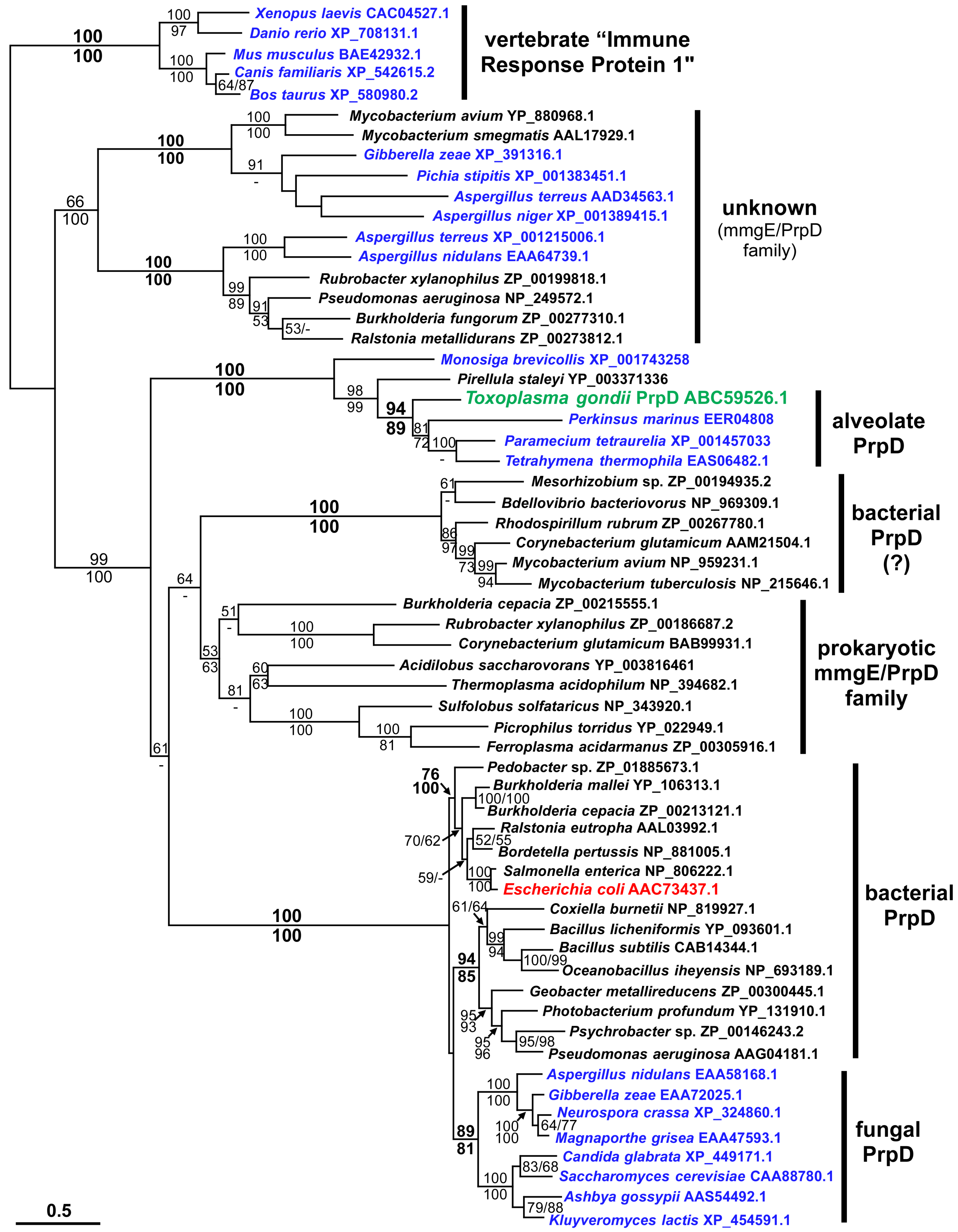

Xenopus laevis CAC04527.1
Danio rerio XP_708131.1
Mus musculus BAE42932.1
Canis familiaris XP_542615.2
Bos taurus XP_580980.2
vertebrate "Immune Response Protein 1"
Mycobacterium avium YP_880968.1
Mycobacterium smegmatis AAL17929.1
Gibberella zeae XP_391316.1
Pichia stipitis XP_001383451.1
Aspergillus terreus AAD34563.1
Aspergillus niger XP_001389415.1
Aspergillus terreus XP_001215006.1
Aspergillus nidulans EAA64739.1
Rubrobacter xylanophilus ZP_00199818.1
Pseudomonas aeruginosa NP_249572.1
Burkholderia fungorum ZP_00277310.1
Ralstonia metallidurans ZP_00273812.1
unknown
(mmgE/PrpD family)
Monosiga brevicollis XP_001743258
Pirellula staleyi YP_003371336
Toxoplasma gondii PrpD ABC59526.1
Perkinsus marinus EER04808
Paramecium tetraurelia XP_001457033
Tetrahymena thermophila EAS06482.1
alveolate PrpD
Mesorhizobium sp. ZP_00194935.2
Bdellovibrio bacteriovorus NP_969309.1
Rhodospirillum rubrum ZP_00267780.1
Corynebacterium glutamicum AAM21504.1
Mycobacterium avium NP_959231.1
Mycobacterium tuberculosis NP_215646.1
bacterial PrpD (?)
Burkholderia cepacia ZP_00215555.1
Rubrobacter xylanophilus ZP_00186687.2
Corynebacterium glutamicum BAB99931.1
Acidilobus saccharovorans YP_003816461
Thermoplasma acidophilum NP_394682.1
Sulfolobus solfataricus NP_343920.1
Picrophilus torridus YP_022949.1
Ferroplasma acidarmanus ZP_00305916.1
prokaryotic mmgE/PrpD family
Pedobacter sp. ZP_01885673.1
Burkholderia mallei YP_106313.1
Burkholderia cepacia ZP_00213121.1
Ralstonia eutropha AAL03992.1
Bordetella pertussis NP_881005.1
Salmonella enterica NP_806222.1
Escherichia coli AAC73437.1
Coxiella burnetii NP_819927.1
Bacillus licheniformis YP_093601.1
Bacillus subtilis CAB14344.1
Oceanobacillus iheyensis NP_693189.1
Geobacter metallireducens ZP_00300445.1
Photobacterium profundum YP_131910.1
Psychrobacter sp. ZP_00146243.2
Pseudomonas aeruginosa AAG04181.1
bacterial PrpD
Aspergillus nidulans EAA58168.1
Gibberella zeae EAA72025.1
Neurospora crassa XP_324860.1
Magnaporthe grisea EAA47593.1
Candida glabrata XP_449171.1
Saccharomyces cerevisiae CAA88780.1
Ashbya gossypii AAS54492.1
Kluyveromyces lactis XP_454591.1
fungal PrpD
0.5

Figure S3

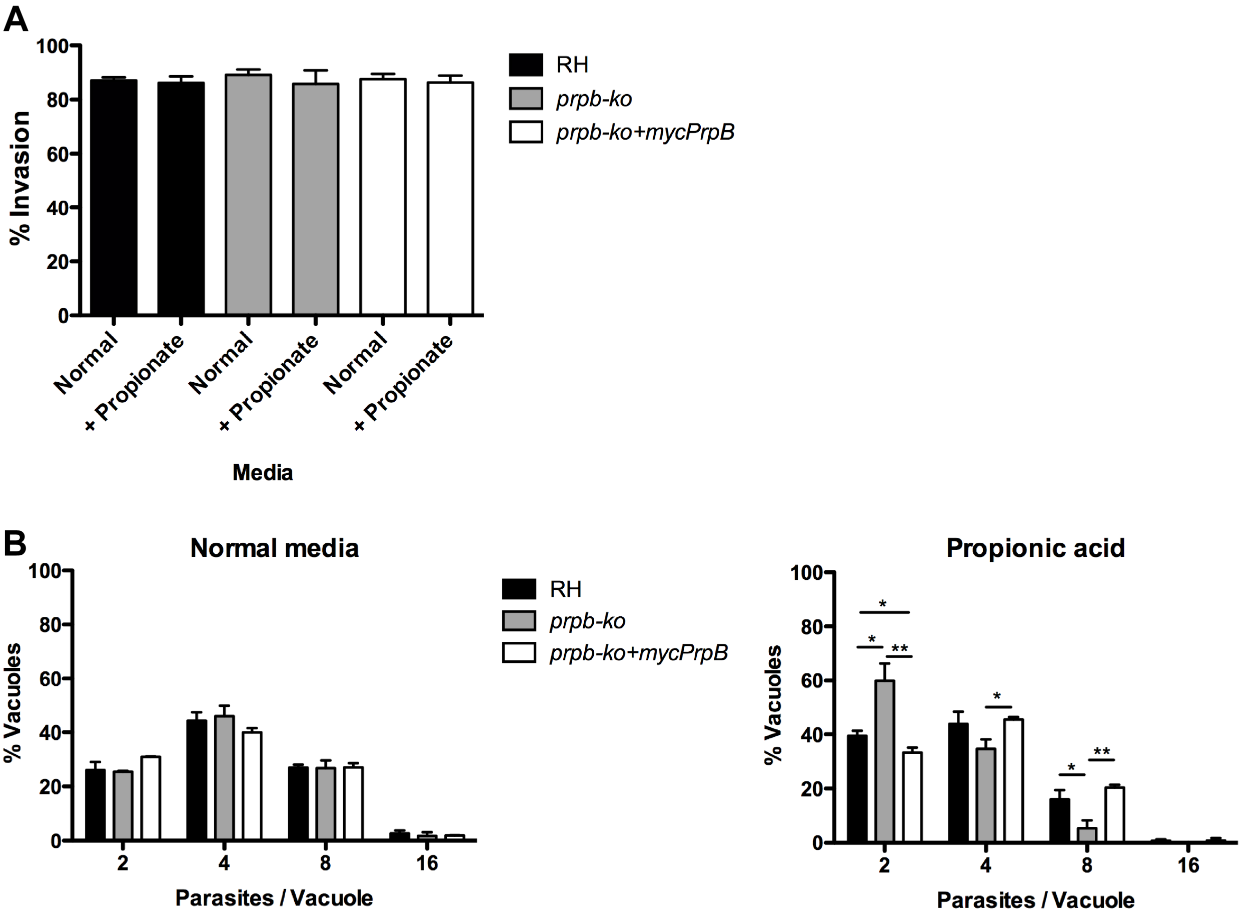
A
% Invasion
100
80
60
40
20
0
Normal
+ Propionate
Normal
+ Propionate
Normal
+ Propionate
Media
RH
prpb-ko
prpb-ko+mycPrpB
B
Normal media
% Vacuoles
100
80
60
40
20
0
2
4
8
16
Parasites / Vacuole
RH
prpb-ko
prpb-ko+mycPrpB
Propionic acid
% Vacuoles
100
80
60
40
20
0
*
*
**
*
*
**
2
4
8
16
Parasites / Vacuole

Figure S4

A

```
           1        10        20        30        40        50        60        70        80        90       100       110       120       130
MsPrpB                                                          MGSTVSAAQKRKAFRDALDSGRLQRMPGAFSPLVAKSLTDIGFDGIYVSGAVLSADLGLPDIGLTTLTEVVTRSAQIAS
TgPrpB     MTIEFDVPKSFCFDFRKECLEPLSVSTSFFVALPRRLPVLVSAFRLTTSLHSHSMASRAPHAGQRLRSLMQKKCVMLPGAYNGLTARLAAEAGFEGVYVSGAALSACQGVPDIGILGLEDFTRVISQAAS
Consensus  .....................................................mgSmaSaAqhagarrraLdqgrcqr$PGA%ngLtArlaa#aGF#G!YVSGAaLSAdqGlPDIGilgLe#ftrriaQaAS

           131      140       150       160       170       180       190       200       210       220       230       240       250       260
MsPrpB     ATDLFTLVDADTGFGEPMSAARTVTLLEDAGLAGCHLEDQVNPKRCGHLDGKAVVPAEDMVKRLRAAVAARRD---PNFIICARTDAAGIEGIDAAIERAKTYADAGADLIFTEALSTPEEFAAFRAAVD
TgPrpB     VTSLPVLADADTGFGGPEMVRRTVFAYNQAGAAGLHIEDQRLPKKCGHLEGKQLVSIEEMEEKIKAAAAASQDCSNGDFIICARTDARSVDGLDAAVERAVRYTAAGADMLFPEGLETEEEFQAFAHALA
Consensus  aTdLftLaDADTGFGePemaaRTVfal##AGaAGcHiEDQrnPKrCGHL#GKalVpaE#MeerirAAaAArrD...g#FIICARTDAag!#GiDAA!ERAkrYaaAGAD$iFpEaLeTeEEFaAFaaAla

           261      270       280       290       300       310       320       330       340       350       360      369
MsPrpB     TP----------LLANMTEFGKSELVNARELADIGYNVVIYPVTTLRLAMHAVEIGMREVFDAGTQSGLLGSMQHRSRLYELLRYADYNQFD----SDIYNFAVQEVRA
TgPrpB     VLPGKAPFGGPYLLANMTEFGKTPIMELSTFEGLGYHCVIYPVSPLRVAMKSVKGMLVDLRKNGSVGHSLEKMYTRQELYSTLHYRPEGTWTYPSASVCMDKAVEDTEA
Consensus  tl..........LLANMTEFGKseim#areladiGYncVIYPVspLRlAMhaVegg$r#lrdaGsqgglLekMqhRqrLYelLrYadenqfd....Sdcm#kAV##trA
```

Figure S5A

2-methyl-cis-aconitate (putative): *m/z* = 187.0246 , Rt = 18.8 min (red = prpB KO , black = Rh WT)

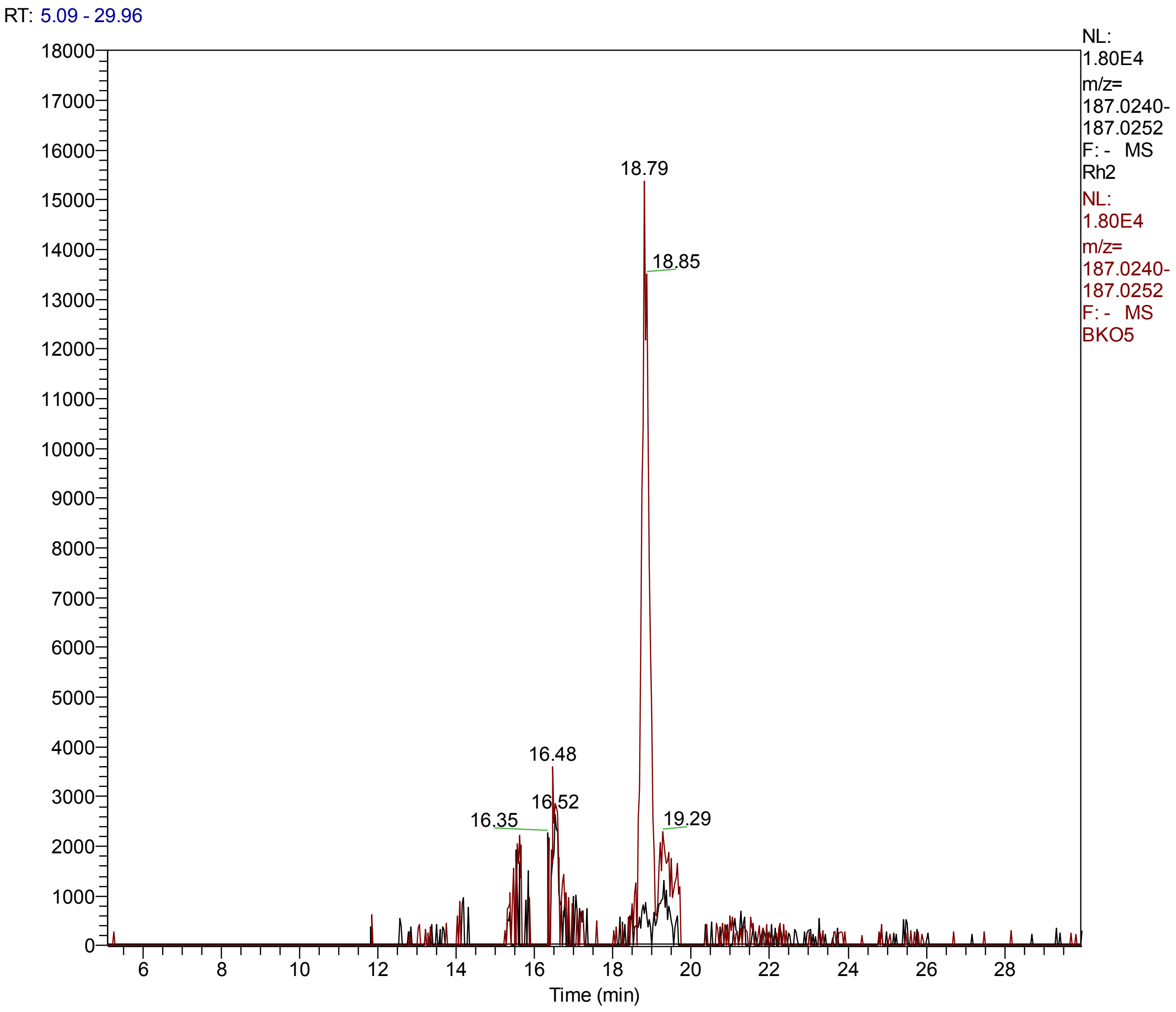

Figure S5B

2-methyl-(iso)citrate (both isomers): *m/z* = 205.0353 , Rt = 19.4 min (red = prpB KO , black = Rh WT)

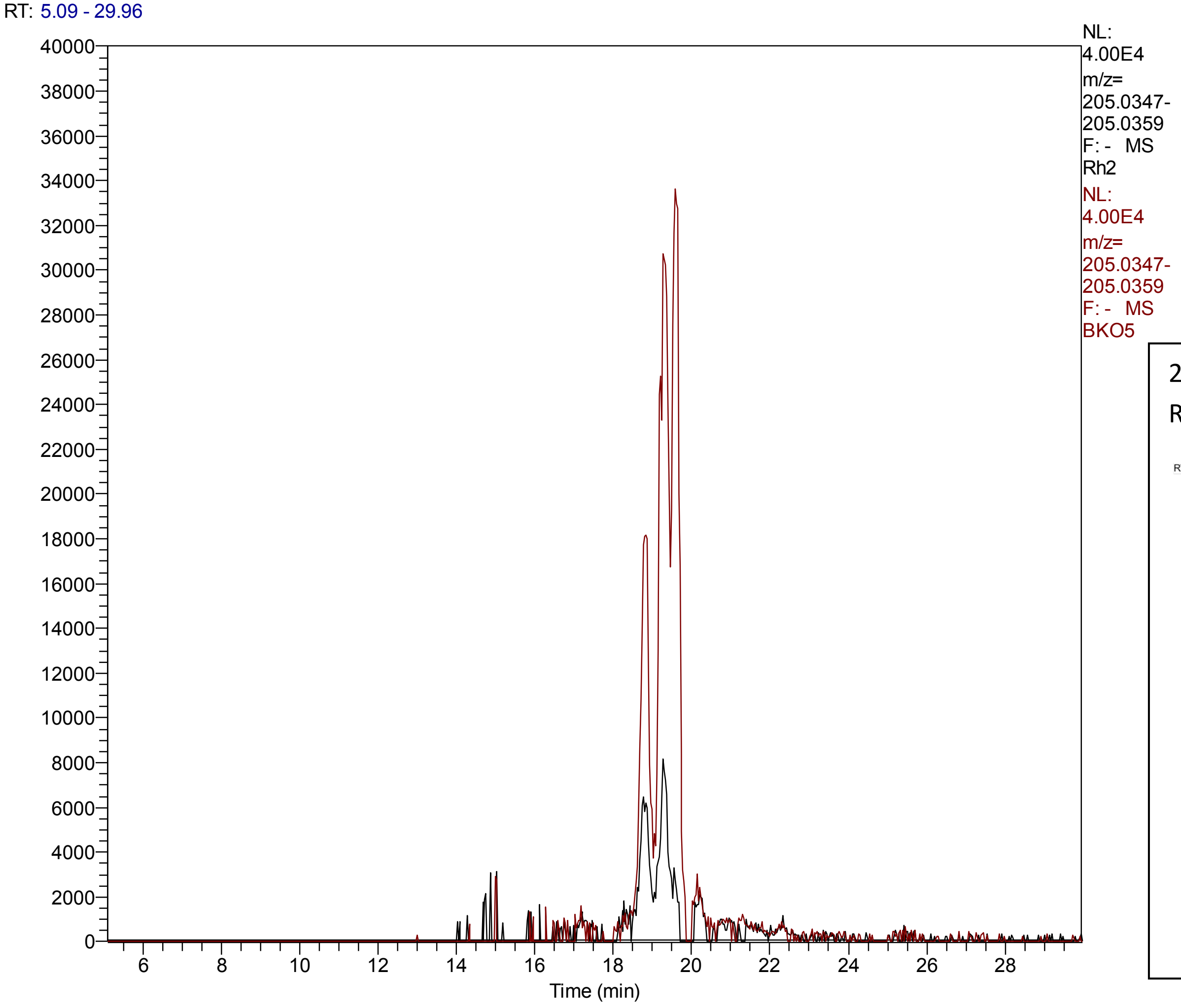

2-methylcitrate authentic standard : *m/z* = 205.0353, Rt = 19.4 min

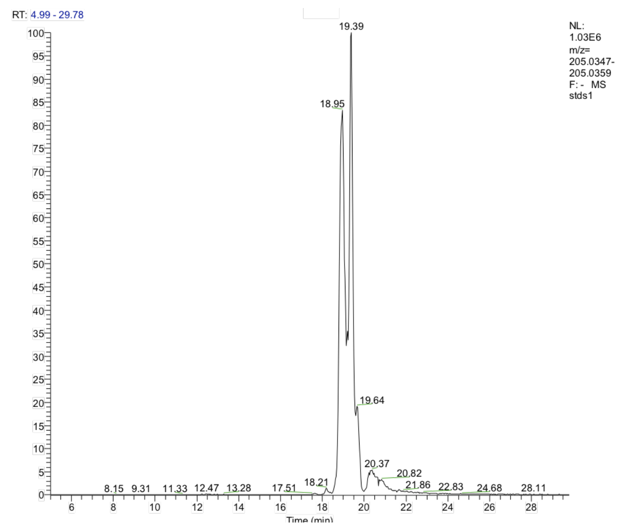

Figure S5C

(iso)citrate (both isomers): *m/z* = 191.0198 , Rt = 20.1 min (red = prpB KO , black = Rh WT)

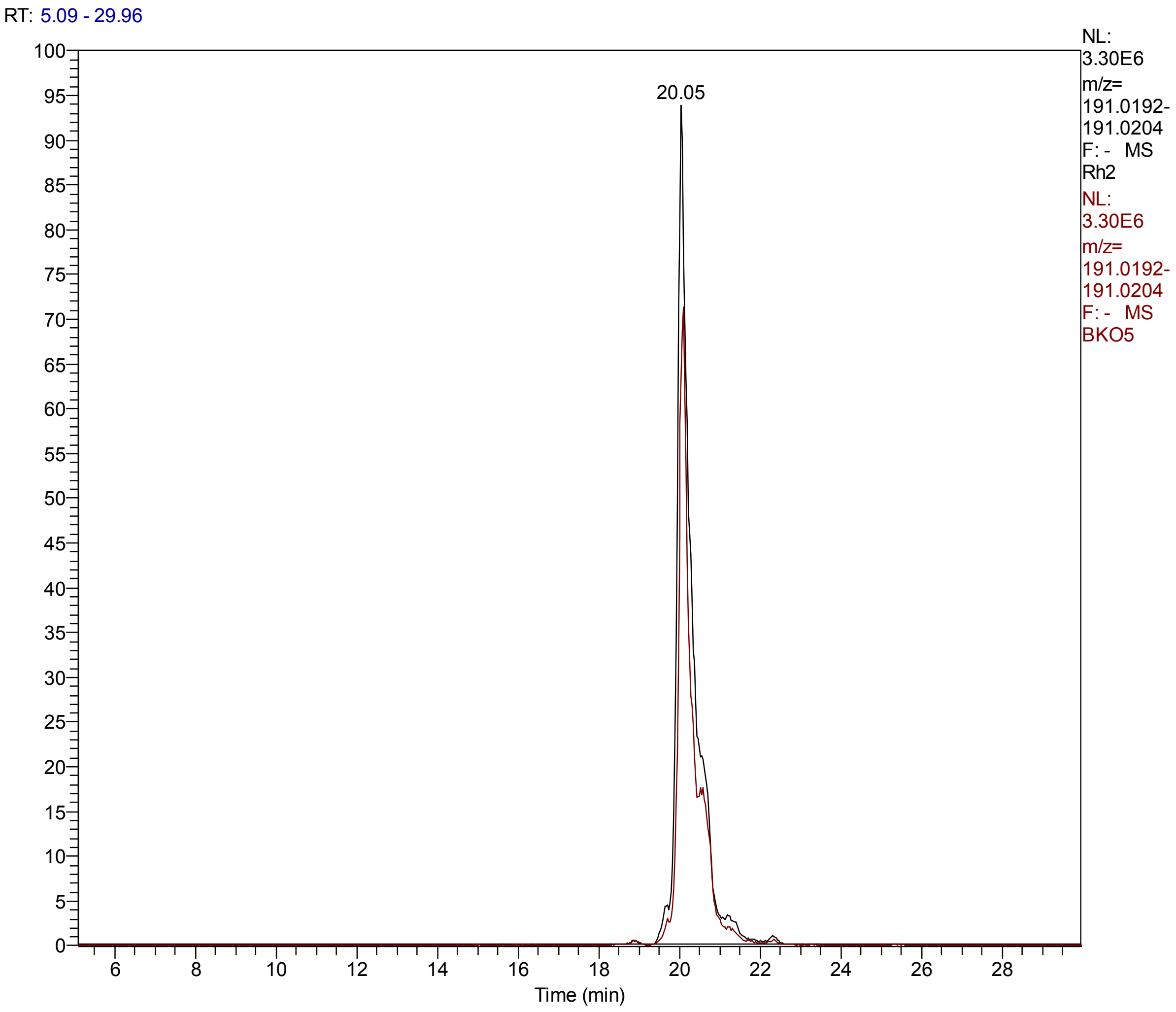

Figure S5D

Succinate: *m/z* = 117.0194 , Rt = 17 min (red = prpB KO , black = Rh WT)

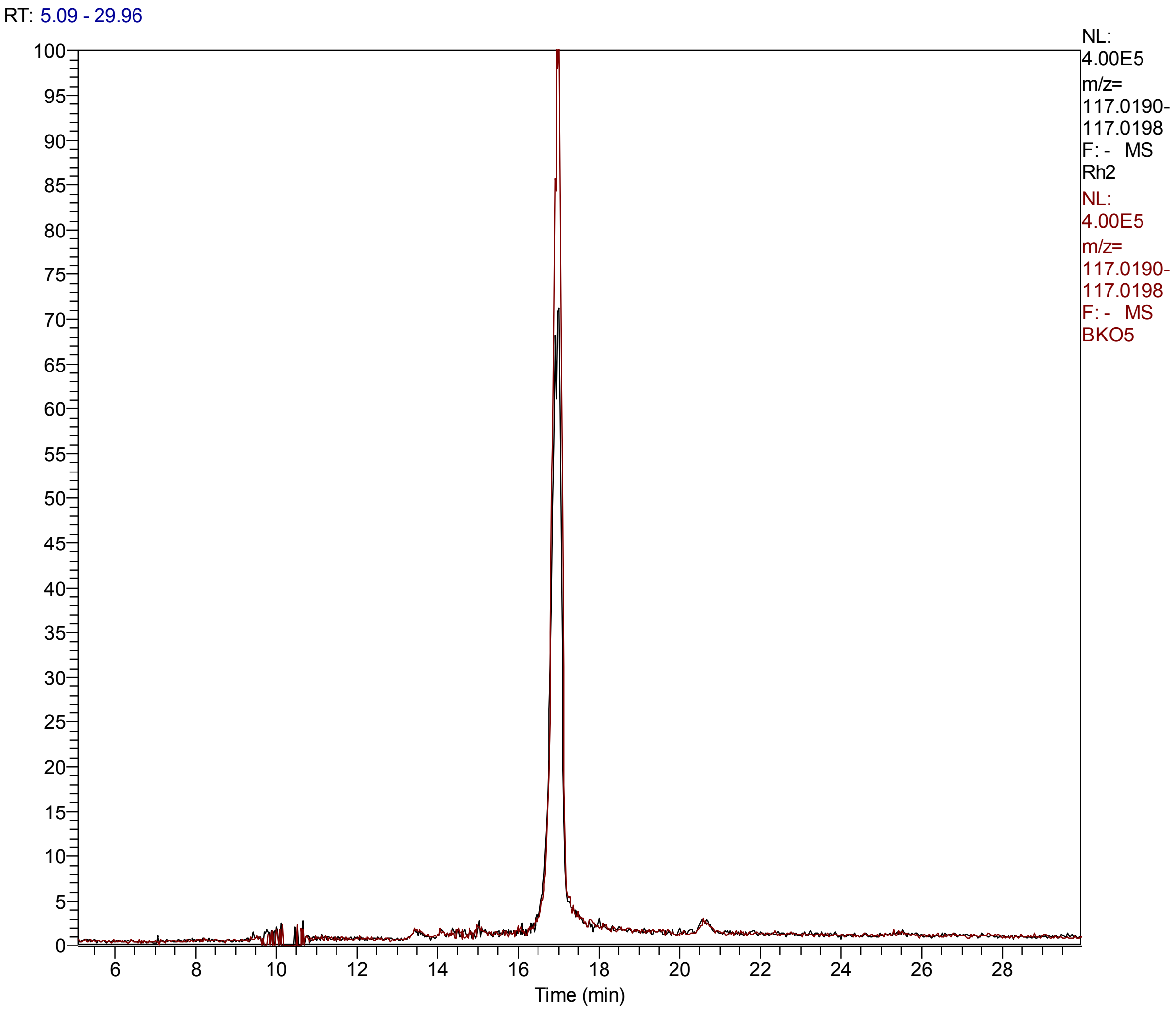

Figure S5E

2-oxoglutarate: *m/z* = 145.0142, Rt = 17.4 min (red = prpB KO , black = Rh WT)

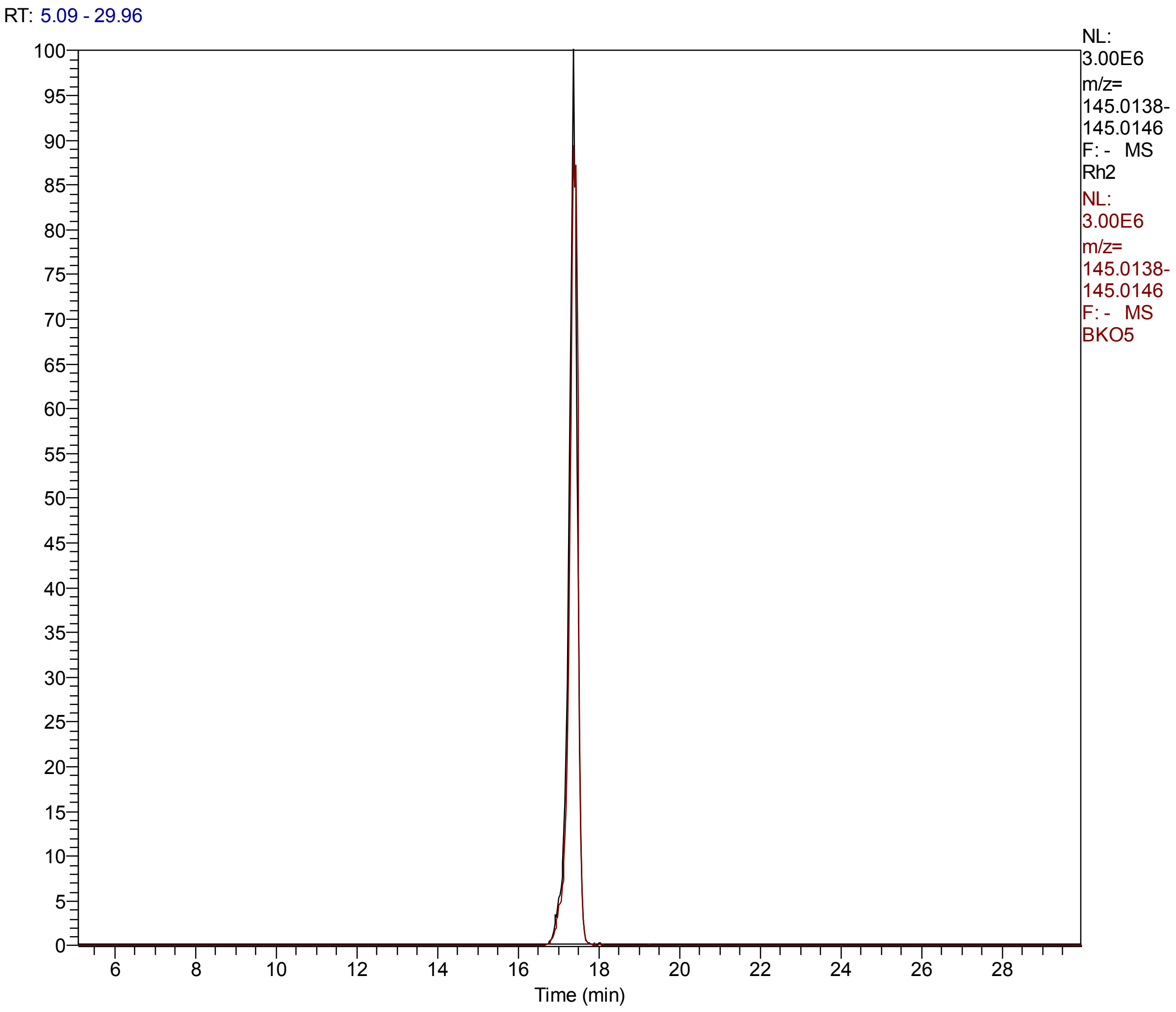

Figure S5F

Pyruvate: *m/z* = 87.0089, Rt = 8 min (red = prpB KO , black = Rh WT)

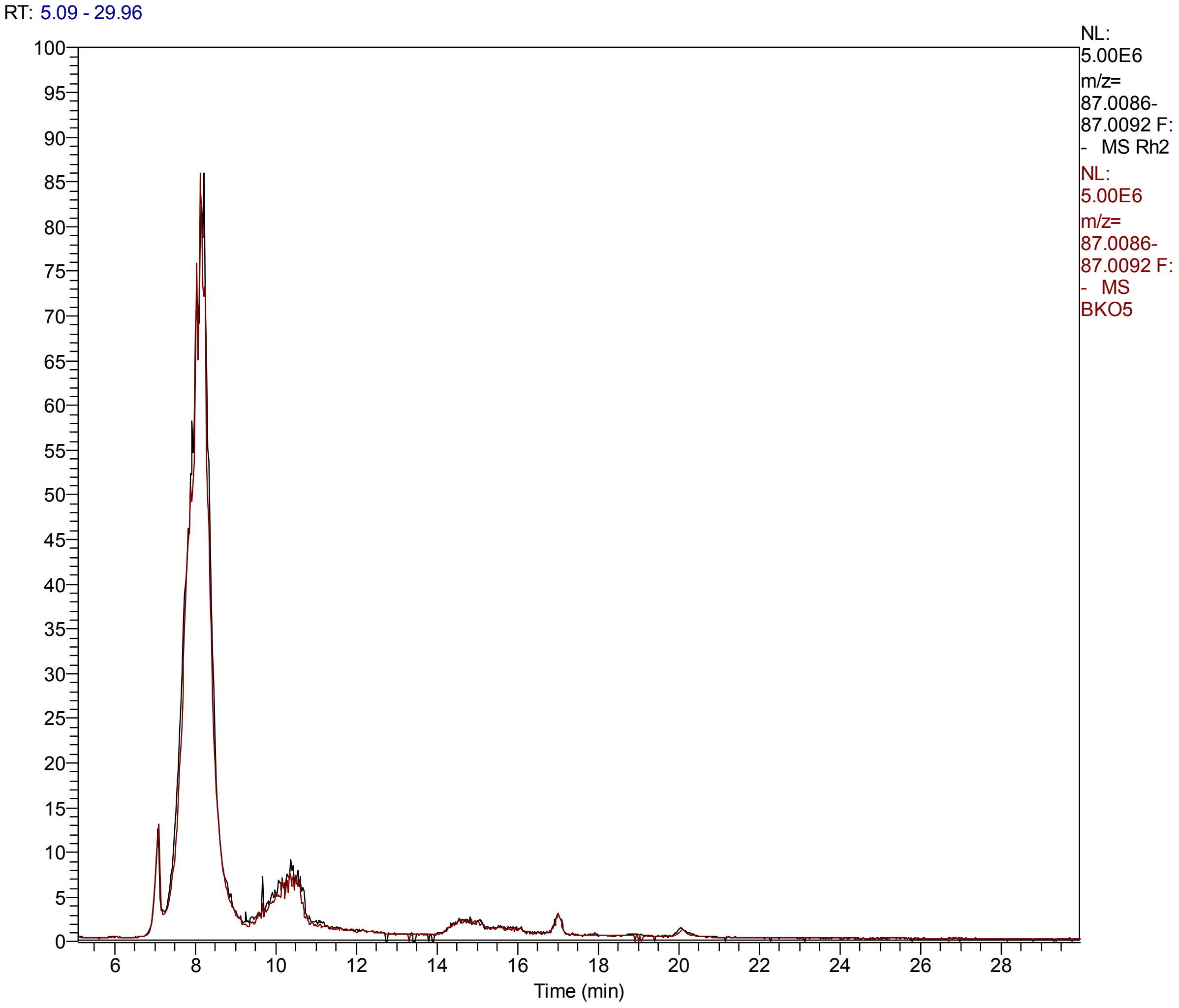

Figure S5G

Valine: *m/z* = 118.0862, Rt = 13.5 min (red = prpB KO , black = Rh WT)

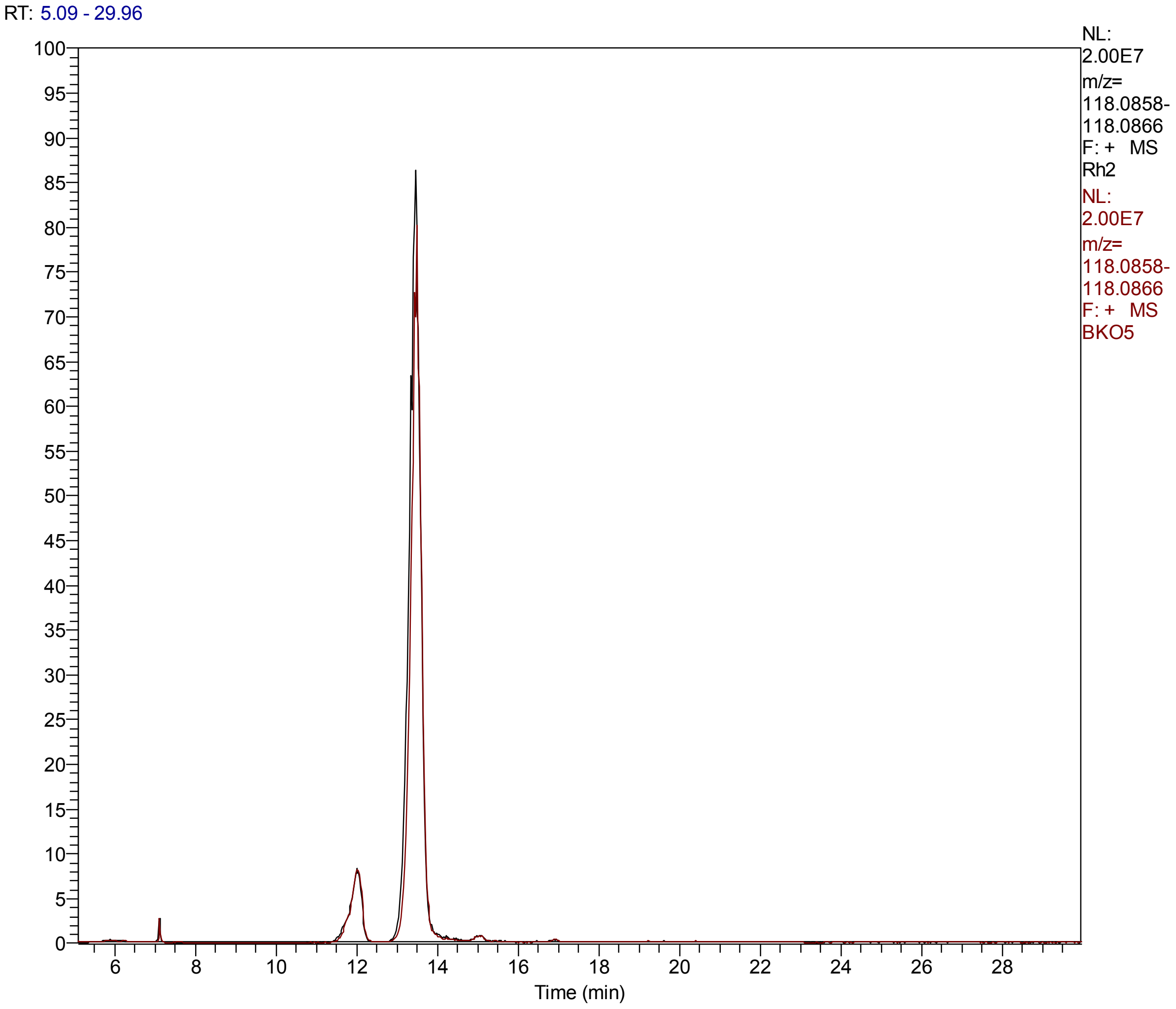

Figure S5H

L-(iso)leucine (both isomers): *m/z* = 132.1019, Rt = 12 min (red = prpB KO , black = Rh WT)

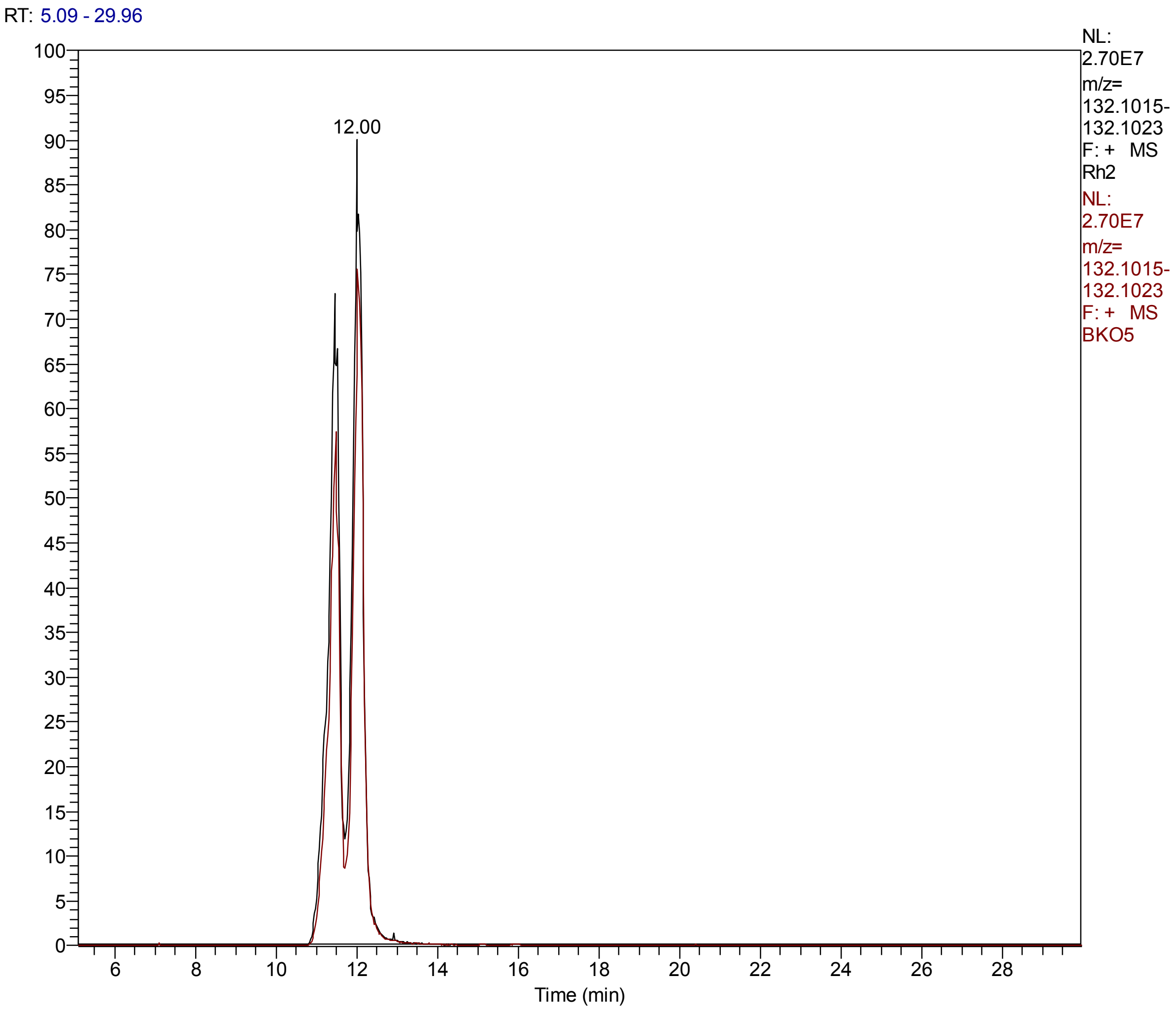

Table S1: Primer list

Name	Sequence 5' -> 3'	R.E. site	Purpose
1362	CCGGAATTCCCTTTTTCGACAAAATGTCCCGAAATCCTTTTGTTTTGATTTCC	EcoRI	*Fwd* - PrpB Ty C-term epitope tagging in pT8MLCTy-HX
1363	GGGCCTGCAGGGGCCTCGGTATCTTCCACGG	SbfI	*Rev* - PrpB Ty C-term epitope tagging in pT8MLCTy-HX, Figure 3A primer B
1567	CCGCTGCAGATTGAGTTCGATGTCCCGAAATCC	PstI	*Fwd* - myc PrpB N-term epitope tagging in pT8MycGFPMyoAtail-HX
1568	GGCTTAATTAACTAGGCCTCGGTATCTTCCACGGC	PacI	*Rev* - myc PrpB N-term epitope tagging in pT8MycGFPMyoAtail-HX
1411	GGCGAATTCCCTTTTTCGACAAAATGACTCAAAGTACTTCAGCTGACAC	EcoRI	*Fwd* - PrpD Ty C-term epitope tagging in pT8MLCTy-HX
1389	CCGCTGCAGCCTCAATTGGCGGAAGCTCATTTAACTC	NsiI	*Rev* - PrpD Ty C-term epitope tagging in pT8MLCTy-HX
2080	CCGGAATTCATGTTTTCAGTGCTACAAATTACCTGTCG	EcoRI	*Fwd* - N-terminal PrpE Ty C-term epitope tagging in pT8-GFP-Ty-HX
2301	GGCATGCATTCGGTACCACTGGCATATAGATCAG	NsiI	*Rev* - N-terminalPrpE Ty C-term epitope tagging in pT8-GFP-Ty-HX
1429	GGCGAATTCCCTTTTTCGACAAAATGGCGGCTGCCGCTAGAAC	EcoRI	*Fwd* - PrpC Ty C-term epitope tagging in pT8MLCTy-HX
1419	GGCCCTGCAGGGAGCCGACTTCTGGACTGC	SbfI	*Rev* - PrpC Ty C-term epitope tagging in pT8MLCTy-HX
1428	GGCGAATTCCCTTTTTCGACAAAATGACCATTGAGTTCGATGTCCC	EcoRI	*Fwd* - Express recombinant PrpB to generate antibodies, Figure 3A primerA
1363	GGGCCTGCAGGGGCCTCGGTATCTTCCACGG	SbfI	*Rev* - Express recombinant PrpB to generate antibodies
1460	GCCGGTACCCTTGGAATGTTTCCACTTC	KpnI	*Fwd* - Amplify 5'UTR of PrpB to generate KO in pTub5CAT
1461	GGGCTCGAGTTCACGACGGAAGGTG	XhoI	*Rev* - Amplify 5'UTR of PrpB to generate KO in pTub5CAT
1462	GGGGGATCCAACCAGTGTGCAAGAGTACC	BamHI	*Fwd* - Amplify 3'UTR of PrpB to generate KO in pTub5CAT
1463	GGGGCGGCCGCCTCCTGTGGGGAAAGCG	NotI	*Rev* - Amplify 3'UTR of PrpB to generate KO in pTub5CAT
2073	GCCGCCAGGGTAGTTTGATAAGC		Figure 3A,B primer C
767A	CAGTTTCTTTATAATGGGGC		Figure 3A,B primer D
2169	CCGGGCATGCAGGAGAAAAAAATCACTGGA		Figure 3A,B primer E
2072	CTGAGAGCTCCATGACGGGTTCTGC		Figure 3A,B primer F
2657	CCGGGTACCGTACCGCCACTCCTGATGGG	KpnI	*Fwd* - Amplify 5'UTR of BCAT to generate KO in pTub5HXGPRT
2658	CCGCCTCGAGCGAAAAGCTAGTCACGAAAACAATGAAGG	XhoI	*Rev* - Amplify 5'UTR of BCAT to generate KO in pTub5HXGPRT
2659	CCGGGATCCCTCCCTAGAACCAGTCGAATTAGTCATG	BamHI	*Fwd* - Amplify 3'UTR of BCAT to generate KO in pTub5HXGPRT
2660	CCGGCGGCCGCTCTTGTTGGCTTTCGGATAAAATCG	NotI	*Rev* - Amplify 3'UTR of BCAT to generate KO in pTub5HXGPRT
1686	CCGGAATTCCCTTTTTCGACAAAATGCGCCTGTGGAAGAGTCATG		Figure 5A,B primer A
1687	GGCATGCATCACATTCCTGCATGAAGTGATGGGGTAC		Figure 5A,B primer B
2694	CCGGTACGATGAGCTTCTGC		Figure 5A,B primer C
2074	CCGTAGTCTTCAATGGGTTTGGACGC		Figure 5A,B primer D
2581	GCCCACGACAGCAGACAACTTTCC		Figure 5A,B primer E
2695	CCGTCAACTGAACTGCGTCGAG		Figure 5A,B primer F
1686	CCGGAATTCCCTTTTTCGACAAAATGCGCCTGTGGAAGAGTCATG	EcoRI	*Fwd* - BCAT-Ty C-term epitope tagging in pT8MLCTy-HX
1687	GGCATGCATCACATTCCTGCATGAAGTGATGGGGTAC	NsiI	*Rev* - BCAT-Ty C-term epitope tagging in pT8MLCTy-HX
2772	CGCAAGCTTCAATGACCATTGAGTTCGATGTCCCG	HindIII	*Fwd* - Amplify full length TgPrpB to clone in pMV261 vector for M.smegmatis complementation
2788	CGCAAGCTTCAATGTCTCACAGCATGGCGTCTCGTGC	HindIII	*Fwd* - Amplify short TgPrpB to clone in pMV261 vector for M.smegmatis complementation
2789	GCGAAGCTTGCTAGGCCTCGGTATCTTCCAC	HindIII	*Rev* - Amplify TgPrpB to clone in pMV261 vector for M.smegmatis complementation
4227	GCCAAGCTTCAATGTCGACCGTTGGCACC	HindIII	*Fwd* - Amplify M.smegmatis icl1 to clone in pMV261 vector for M.smegmatis complementation
4228	GCCAAGCTTCTCAGTGGAACTGACCCTC	HindIII	*Rev* - Amplify M.smegmatis icl1 to clone in pMV261 vector for M.smegmatis complementation

Table S2: Virulence *in vivo* of *prprb-ko*

	Day 0	Day 5 post-infection	Day 6 post-infection
**WT**	5 mice injected intraperitoneally with ~30-50 RH parasites	mice presented signs of illness (bristle hair)	Mice very ill (bristle hair, loss of movement and muscular tonicity) All mice sacrificed
prpb-ko	5 mice injected intraperitoneally with ~30-50 *prpb-ko* parasites	mice presented signs of illness (bristle hair)	Mice very ill (bristle hair, loss of movement and muscular tonicity) All mice sacrificed

Table S3 Raw peak heights for prpB knockout vs wild type

Metabolite	Mass[a]	RT (min)	Wild-type (RH)			*prpb-ko*			*prpb-ko*+mycPrpB		
			Rh1	Rh2	Rh3	BKO4	BKO5	BKO6	BKOmycB7	BKOmycB8	BKOmycB9
**2-methyl-cis-aconitate[b]**	188.0318	18.95	1,130	1,306	902	14,090	15,375	12,128	1,065	917	988
**2-Methyl (iso) citrate[c]**	206.0426	19.22	10,094	8,157	4,697	39,312	33,650	39,317	5,367	3,448	5,232
**Succinate**	118.0266	16.95	373,897	284,867	252,786	436,766	407,955	361,165	402,961	307,985	363,726
**(iso)Citrate[c]**	192.0271	20.07	2,882,673	3,098,548	2,678,980	2,443,727	2,356,873	2,038,751	1,856,598	1,451,357	1,620,305
**2-Oxo glutarate**	146.0215	17.36	3,079,291	3,013,277	2,542,724	2,713,612	2,681,474	2,151,063	1,136,608	882,144	1,112,021
**Pyruvate**	88.0162	8.05	5,085,228	4,300,972	4,558,859	4,026,943	4,297,195	3,500,726	2,297,306	1,201,990	1,653,249
**L-Valine**	117.0790	13.46	19,535,416	17,294,672	14,069,846	15,197,135	16,047,099	12,763,604	19,258,144	12,840,328	19,279,074
**L-(iso) Leucine[c]**	131.0947	12.03	29,544,218	24,335,600	23,144,030	20,967,488	20,412,828	18,046,694	22,727,636	17,158,894	21,958,132

a) Mass corrected by addition of the mass of one proton to the m/z detected in negative mode (Valine and Leucine were detected in positive mode, and therefore corrected by subtraction of the mass of one proton).
b) Putatively identified by exact mass and calculated retention time (no authentic standard available)
c) Mixture of isomers. Peaks not fully separated by LC.
